# Thermosensitive Exosome–Liposome Hybrid Nanoparticle‐Mediated Chemoimmunotherapy for Improved Treatment of Metastatic Peritoneal Cancer

**DOI:** 10.1002/advs.202000515

**Published:** 2020-07-29

**Authors:** Qijun Lv, Lili Cheng, Yao Lu, Xiaoge Zhang, Yizhen Wang, Junfeng Deng, Jiangbing Zhou, Bo Liu, Jie Liu

**Affiliations:** ^1^ Department of General Surgery The Third Affiliated Hospital of Sun Yat‐sen University Guangzhou 510120 China; ^2^ School of Biomedical Engineering Sun Yat‐sen University Guangzhou Guangdong 510006 China; ^3^ Departments of Neurosurgery and of Biomedical Engineering Yale University New Haven CT 06510 USA

**Keywords:** chemoimmunotherapy, exosomes, hyperthermic intraperitoneal chemotherapy, metastatic peritoneal carcinoma, thermosensitive liposomes

## Abstract

Metastatic peritoneal carcinoma (mPC) is a deadly disease without effective treatment. To improve treatment of this disease, a recently developed hyperthermic intraperitoneal chemotherapy (HIPEC) has emerged as the standard of care. However, the efficacy of this approach is limited by inefficient drug penetration and rapidly developed drug resistance. Herein, a nanotechnology approach is reported that is designed to improve drug delivery to mPC and to augment the efficacy of HIPEC through delivery of chemoimmunotherapy. First, the drug delivery efficiency of HIPEC is determined and it is found that chemotherapy agents cannot be efficiently delivered to large tumors nodules. To overcome the delivery hurdle, genetically engineered exosomes‐thermosensitive liposomes hybrid NPs, or gETL NPs, are then synthesized, and it is demonstrated that the NPs after intravenous administration efficiently penetrates into mPC tumors and releases payloads at the hypothermia condition of HIPEC. Last, it is shown that, when granulocyte‐macrophage colony‐stimulating factor (GM‐CSF) and docetaxel are co‐delivered, gETL NPs effectively inhibit tumor development and the efficacy is enhanced when HIPEC is co‐administered. The study provides a strategy to improve drug delivery to mPCs and offers a promising approach to improve treatment of the disease through combination of locoregional delivery of HIPEC and systemic delivery of chemoimmunotherapy via gETL NPs.

## Graphical abstract

In this study, a CD47 over expressed exosomes–liposomes hybrid nanoparticle (gETL NP) delivery system is developed for granulocyte‐macrophage colony‐stimulating factor (GM‐CSF) and docetaxel co‐delivery. Combined with standard hyperthermic intraperitoneal chemotherapy (HIPEC) therapy to metastatic peritoneal carcinoma (mPC), the gETL NP achieves HIPEC‐mediated chemotherapy and CD47‐ and GM‐CSF‐mediated immunotherapy.

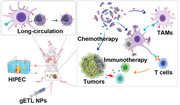

## 1 Introduction

Metastatic peritoneal carcinoma (mPC) is a fatal disease without effective treatment.^[^ ^1^, ^2^ ^]^ With the current standard of care, patients with mPC often succumb to the disease within a few months.^[^ ^3^, ^4^ ^]^ To improve treatment of this disease, a new approach, called hyperthermic intraperitoneal chemotherapy (HIPEC), was developed, which involves intraperitoneal drug filling with heating.^[^ ^5^, ^6^, ^7^, ^8^, ^9^, ^10^ ^]^ Unfortunately, accumulating evidence suggests that the therapeutic benefit of HIPEC is limited by inefficient drug penetration and rapidly developed drug resistance.^[^ ^11^, ^12^, ^13^, ^14^ ^]^ It is showed that patients with abdominal tumor larger than 1 cm in diameter could not be benefited from intraperitoneal chemotherapy.^[^ ^15^, ^16^, ^17^ ^]^ Therefore, further improved treatment of mPC requires development of HIPEC‐compatible drug delivery system that can enhance drug delivery to tumors and enable multimodal combination therapy to overcome the drug resistance.

To improve drug delivery to tumors, emerging nanotechnology is promising. Due to the enhanced permeability and retention (EPR) effect, the use of nanoparticles (NPs) alters the bio‐distribution of payload and results in preferential drug accumulation in tumors.^[^ ^18^ ^]^ However, the targeting effects of most NPs have been limited their short circulation life in the blood.^[^ ^18^ ^]^ To overcome this problem, surface conjugation of polyethylene glycol (PEG) is often used.^[^ ^19^ ^]^ Alternatively, this can be achieved through surface display of CD47, a “self‐protein” that interacts with the signal regulatory protein alpha receptor (SIRP*α*) in phagocytes and thus enables cargos to escape from clearance by the mononuclear phagocytic system (MPS).^[^ ^20^, ^21^, ^22^, ^23^, ^24^ ^]^ In addition to the enhanced blood circulation, the display of CD47 may also promote phagocytosis of tumor cells through competitive binding with SIRP*α*.^[^ ^25^, ^26^, ^27^ ^]^ However, it is known that the functions of macrophages, including phagocytosis, antigen processing, and antigen presentation, depend on M1 macrophages,^[^ ^28^, ^29^, ^30^ ^]^ while the tumor‐associated macrophages (TAMs) are domesticated to M2 phenotypes.^[^ ^31^ ^]^ Therefore, to fully capitalize the effect of CD47‐mediated phagocytosis enhancement and to maximize the capacity of macrophages for cancer therapy, delivery of cytokines, such as Granulocyte‐macrophage colony‐stimulating factor (GM‐CSF), to promote repolarization of macrophages towards M1 is a promising strategy.^[^ ^32^, ^33^, ^34^ ^]^

**Figure 1:**
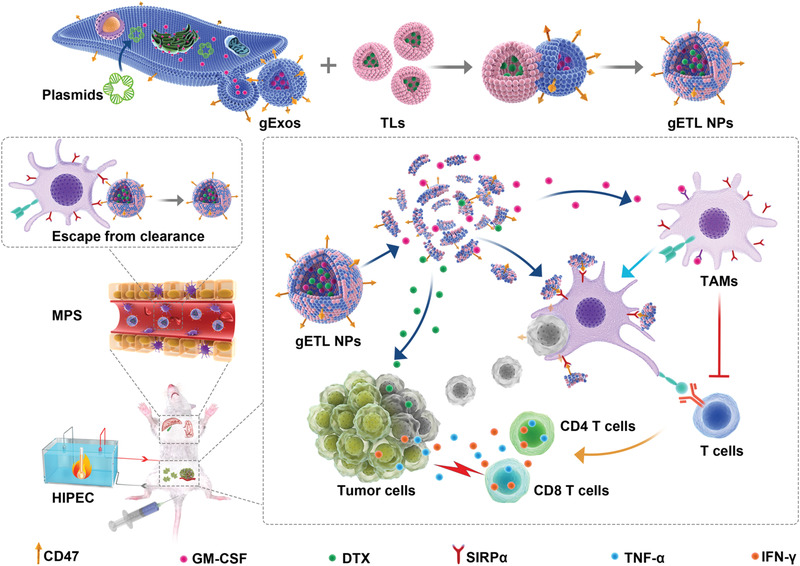
Schematic diagram of the synthesis and application gETL NPs for mPC treatment. Abbreviation: gExos, genetically engineered exosomes; TLs, thermosensitive liposomes; gETL NPs, genetically engineered exosomes‐thermosensitive liposomes hybrid nanoparticles; MPS, mononuclear phagocytic system; TAMs, tumor‐associated macrophages.

In this study, we developed a nanotechnology approach to improve treatment of mPC through combination with HIPEC. First, we characterized the efficiency of drug delivery through HIPEC and found that delivery of chemotherapy agents via HIPEC could not efficiently penetrate large tumor nodules. To overcome the delivery limitation and augment the effect of HIPEC therapy, we then genetically engineered fibroblasts to produce CD47‐expressed exosomes, which were then fused with thermosensitive liposomes. To simplify the nomenclatures, we designated the resulting genetically engineered exosomes‐thermosensitive liposomes hybrid NPs as gETL NPs, and control hybrid NPs without CD47 overexpression as ETL NPs (**Figure** **1**). We demonstrated that gETL NPs after intravenous administration preferentially accumulated in tumors and released payloads in an accelerated rate under the hypothermia condition of HIPEC. Last, we synthesized gETL NPs with loading of GM‐CSF and/or docetaxel (DTX) and characterized them for mPC treatment in both cell line‐derived xenografts (CDX) and patient‐derived tumor xenografts (PDX). We found that treatment with GM‐CSF and DTX‐loaded gETL NPs, or G/D‐gETL NPs, significantly inhibited tumor progression and the antitumor effects were enhanced when HIPEC was combined. Mechanistically, treatment with G/D‐gETL NPs induced macrophage polarization, enhanced macrophage‐mediated tumor cell phagocytosis, and induced cellular apoptosis. This study may provide a new direction for mPC treatment through combination of drug‐loaded gETL NPs with HIPEC.

## 2 Results

### 2.1 Characterization of the Limitation of HIPEC in Drug Delivery

**Figure 2:**
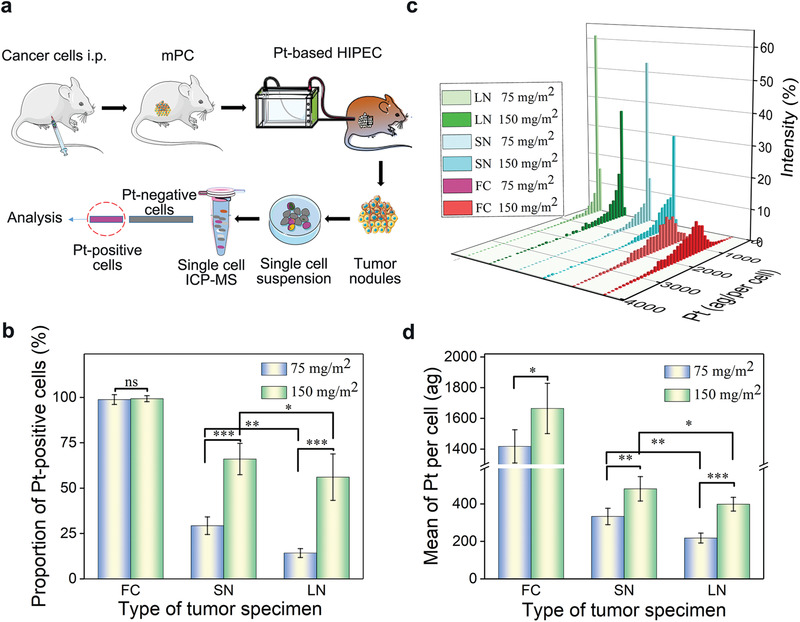
Quantitative evaluation of drug delivery efficiency of HIPEC at the single cell level. a) Schematic illustration of the experiment to evaluate drug delivery efficiency of HIPEC, i.p.: intraperitoneal injection. b) Proportion of Pt‐uptake cells in free tumor cells and tumor nodules post HIPEC procedure at the indicated conditions. c) Distribution of Pt‐uptake cells based on the level of platinum in free tumor cells and tumor nodules. d) Mean weight of platinum in single cells at the indicated conditions. The data was presented as the mean ± SD from three repeated experiments, One‐way ANOVA was used to determine statistical differences (*p < 0.05, **p < 0.01, ***p < 0.001, ns: not significant). Abbreviation, FC: free tumor cells; SN: small tumor nodules (volume < 10 mm^3^); LN: large tumor nodules (volume ≥ 10 mm^3^).

By using murine colorectal carcinoma CT26 cells‐derived mPC model, we studied the efficiency of drug delivery via HIPEC. Oxaliplatin (Pt), a chemotherapy drug that is often used in HIPEC, was administered to tumor bearing mice at 150 or 75 mg m^−2^ (**Figure** **2**a). After 30 min, the mice were euthanized, tumors were isolated and analyzed by inductively coupled plasma mass spectrometry (ICP‐MS) at the single cell level. We found that at the dose of 75 mg m^−2^, 98.8%, 29% and 14% of Pt‐positive cells were found in free tumor cells (FC), small tumor nodules (SN, volume < 10 mm^3^) and large tumor nodules (LN, volume ≥ 10 mm^3^), respectively (Figure 2b); and, at the dose of 150 mg m^−2^, 99.3%, 66%, and 56% Pt‐positive cells were found in FC, SN and LN, respectively (Figure 2b). At both doses, we found that, compared to those in larger sizes, SN had greater drug penetration and uptake (Figure 2b,c) and the amounts of drugs within cells was correlated with the drug concentration in perfusate. For example, at the dose of 75 mg m^−2^, the mean weights of platinum were 1417.8, 313.1, and 217.8 ag per cell in FC, SN, and LN, respectively, while at 150 mg m^−2^ Pt, the mean weights were 1664.8, 480.4, and 398.5 ag per cell in FC, SN, and LN, respectively (Figure 2d). Those results suggest that the drug delivery efficiency of HIPEC to free tumor cells is sufficient for cell killing, while the drug delivery efficiency to tumor nodules of mPC depends on the tumor size and the concentration of drugs administered, and further improved drug delivery to tumor nodules is needed, particularly for those in large sizes.

### 2.2 Synthesis and Characterization of gETL NPs

To improve drug delivery to mPC, we prepared thermosensitive liposomes fused with genetically engineered exosomes. First, we synthesized thermosensitive liposomes through the standard thin‐film hydration method followed by extrusion.^[^ ^35^, ^36^ ^]^ The resulting liposomes were spherical in morphology (Figure S1a, Supporting Information), and had an average diameter of 130.5 nm (Figure S1b, Supporting Information) and zeta potential of −5.4 mV (Figure S1c, Supporting Information). Encapsulation of DTX did not alter the size and morphology of liposomes. DTX‐loaded liposomes released DTX in a temperature‐dependent manner (Figure S1d, Supporting Information). At 41–42 °C that is used in HIPEC, DTX‐loaded liposomes released 75–89% of payload within 10 min; in contrast, only 7% and 9% of DTX was released at 37 °C within 10 and 60 min, respectively (Figure S1d, Supporting Information).

Next, we prepared genetically engineered exosomes. Membrane of exosomes is partially derived from membrane of parent cells. Therefore, display of membrane protein in the surface of exosomes can be achieved through genetically engineering of parent cells.^[^ ^37^ ^]^ In our study, fibroblasts were transduced with lentiviral vectors for overexpression of CD47 and/or GM‐CSF (Figure S2, Supporting Information). Flow cytometry analysis shows that over 63–68% of CD47‐transduced cells expressed CD47, compared to 6–8% in wild type or vehicle control cells (Figure S3a,b, Supporting Information). ELISA analysis suggests that GM‐CSF‐transduced cells produced GM‐CSF at 108 µg mL^−1^, which is significantly greater than the amount produced by control cells (5.5 µg mL^−1^) (Figure S3c, Supporting Information). The genetically engineered fibroblasts were then utilized to produce exosomes, which were collected by ultracentrifugation. Imaging by transmission electron microscopy (TEM) revealed that the genetically engineered exosomes were spherical in morphology (Figure S4a, Supporting Information). Flow NanoAnalyzer determined the size of the exosomes to be 81.4 nm (Figure S4b, Supporting Information). Western blot analysis confirmed the presence of CD63, TSG101, CD9, surface markers of exosomes (Figure S4c, Supporting Information). Flow cytometry analysis found that the expression of CD47 on the genetically engineered exosomes was 13–15 times greater than that on those produced by control cells (Figure S4d, Supporting Information). Analysis by ELISA detected 23.1 pg of GM‐CSF in every 1 µg of genetically engineered exosomes (Figure S4e, Supporting Information).

**Figure 3:**
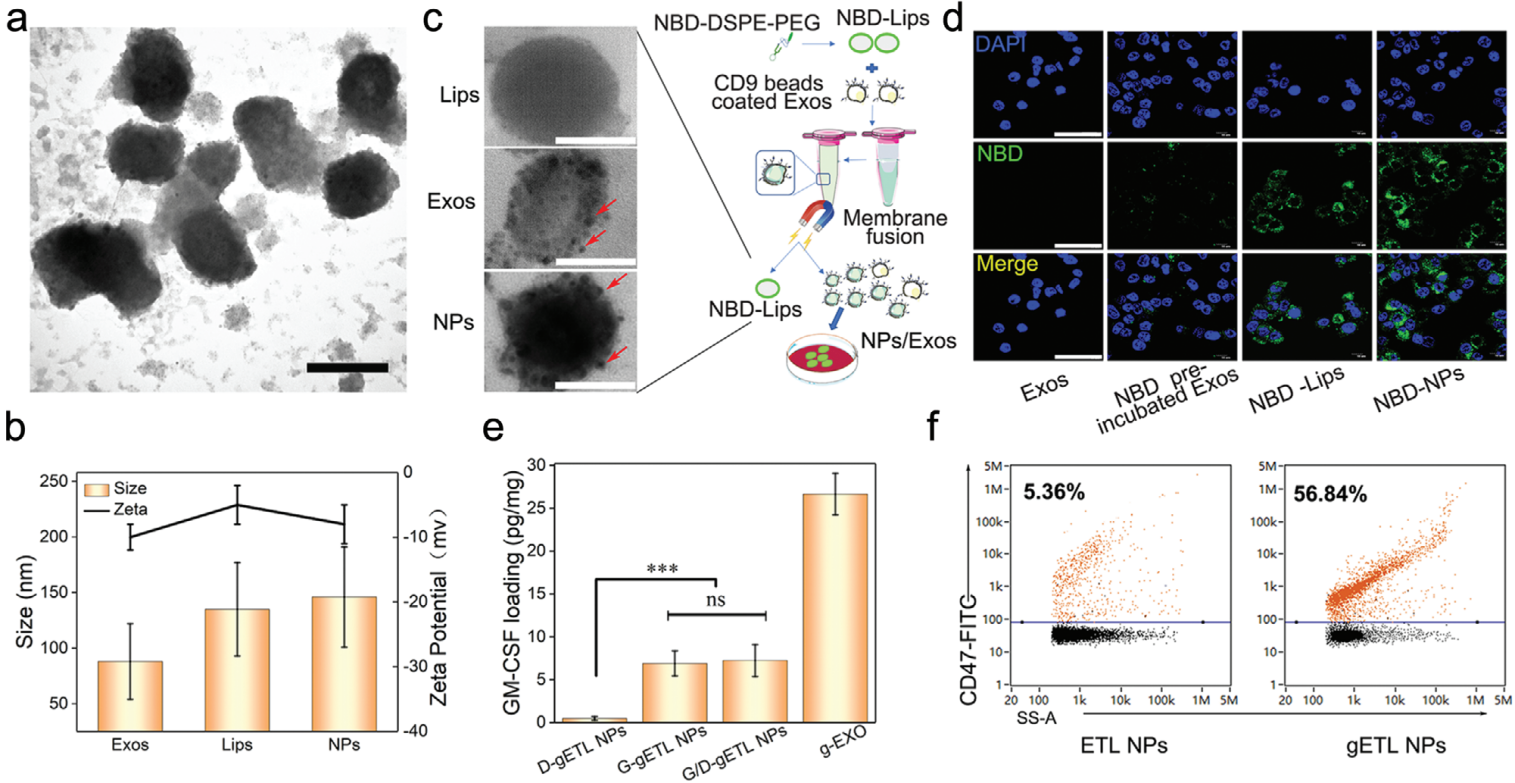
Synthesis and characterization of gETL NPs. a) A representative image of gETL NPs captured by TEM. Scale bar: 100 nm. b) Diameter and Zeta potential of gETL NPs, exosomes and liposomes. c) Schematic diagram of membrane fusion between liposomes and exosomes (right), and representative TEM images of CD9 bead‐labeled vesicles (Lips), exosomes (Exos), and gETL NPs (NPs) (left). Scale bar: 50 nm. d) Representative images of HCT116 cells treated with naïve exosomes, NBD‐DSPE‐PEG_2000_ pre‐incubated exosomes, NBD‐liposomes, or NBD‐NPs. After incubation with the indicated NPs for 2 h, the cells were stained with DAPI and imaged by CLSM. Scale bar: 50 µm. e) Quantification of GM‐CSF in the indicated NPs determined by ELISA. The data were presented as the mean ± SD from three repeated experiments, One‐way ANOVA was used to determine statistical differences. (***p < 0.001, ns: not significant). f) Analysis of the presence of CD47 on the surface of ETL NPs and gETL NPs by Flow NanoAnalyzer.

With thermosensitive liposomes and genetically engineered exosomes, we generated gETL NPs using a previously reported freeze–thaw procedures.^[^ ^38^ ^]^ To confirm the success of fusion, we labeled liposomes with nitrobenzoxadiazole (NBD) through insertion of NBD‐DSPE‐PEG_2000_ and labeled exosomes with CD9 immunomagnetic bead through antigen–antibody reaction. After fusion, using a magnet which pulled down immunomagnetic beads, we sorted out hybrid vesicles and unfused exosomes, which were then analyzed for presence of liposome membrane based on NBD fluorescence using by Flow NanoAnalyzer. Through this method, we found that the fusion efficiency is 95.7% (Figure S5, Supporting Information). TEM analysis showed that gETL NPs were morphologically similar to liposomes (**Figure** **3**a), and had an average diameter of 135.7 nm and Zeta potential of −8.2 mV (Figure 3b). The presence of CD9 beads on the surface of exosomes or gETL NPs was confirmed by TEM (Figure 3c). To further validate the liposome–exosome fusion, gETL NPs, along with non‐fused liposomes and exosomes, were incubated with HCT116 cells. After 2 h, the cells were imaged by confocal laser scanning microscopy (CLSM). Result in Figure 3d show that both gETL NPs and NBD‐labeled liposomes‐treated cells carried strong fluorescence, while weak fluorescence was observed in cells treated with naïve and NBD‐DSPE‐PEG_2000_ pre‐incubated exosomes. We also found that the fluorescence intensity of the gETL NPs treated group was stronger than that of the liposome treated group, suggesting that the inclusion of exosome components promoted the cellular uptake (Figure 3d).

Using the procedures described above, we synthesized GM‐CSF‐ and DTX‐ loaded gETL NPs, or G/D‐gETL NPs, through fusion of DTX‐loaded liposomes with exosomes produced by the CD47/GM‐CSF‐transduced cells. The resulting NPs were characterized for encapsulation of GM‐CSF and DTX as well as the presence of CD47. We found that the loadings of GM‐CSF and DTX in G/D‐gETL NPs were 7.2 pg µg^−1^ and 4.3%, respectively, which were lower than the amount of GM‐CSF in the engineered exosomes (26.65 pg µg^−1^) (Figure 3e) and the amount of DTX in the DTX‐loaded liposomes (5.6%) prior to fusion. To confirm surface presence of CD47, we incubated the G/D‐gETL NPs with FITC‐conjugated CD47 antibody and analyzed by Flow NanoAnalyzer. Results in Figure 3f showed that, similar to those for the genetically engineered cells and exosomes (Figures S3a,b and S4d, Supporting Information), 56.8% gETL NPs were positive for CD47, compared to ∼5% for ETL NPs that were generated using wild type exosomes (Figure 3f; Figure S4d, Supporting Information).

### 2.3 gETL NPs Inhibit Tumor Cell Proliferation Synergistically with HIPEC In Vitro

**Figure 4:**
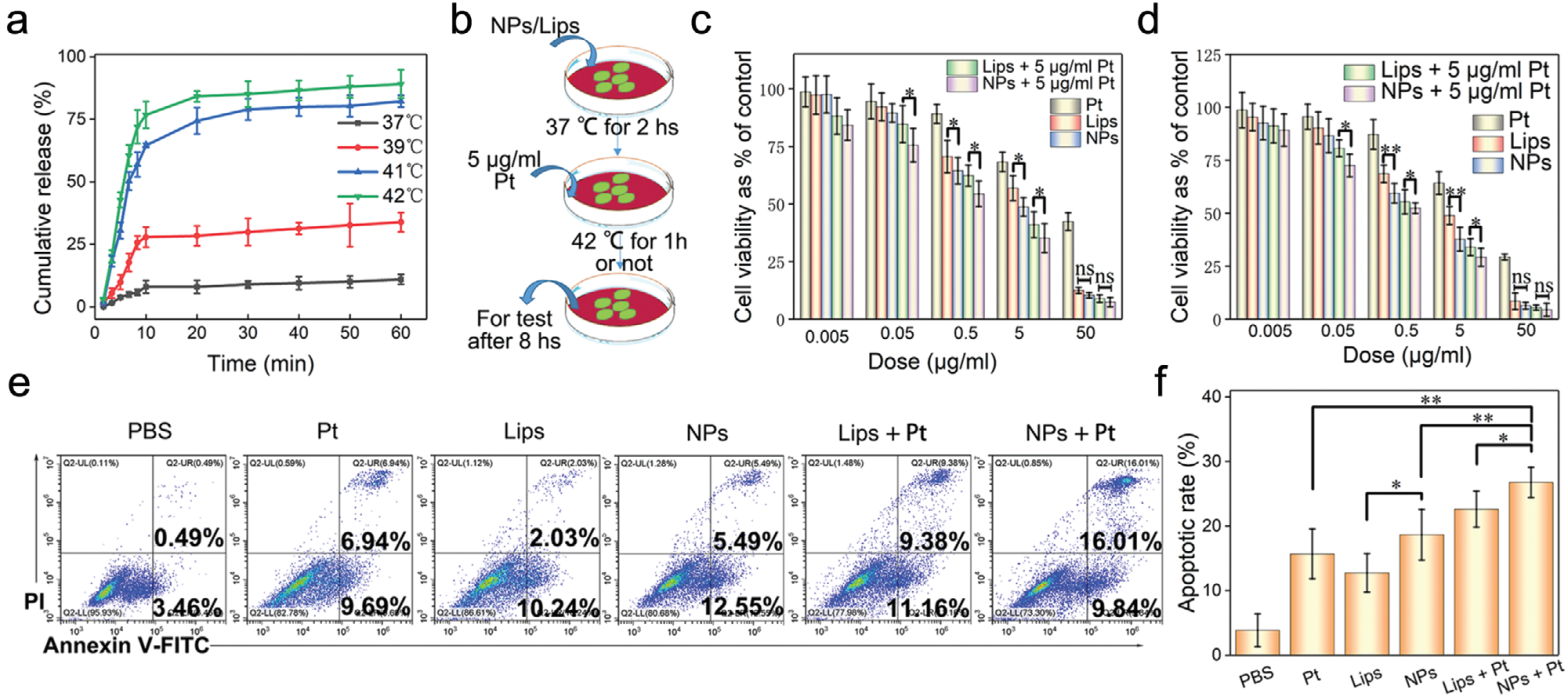
Characterization of the antitumor effect of gETL NPs. a) Characterization of drug release behavior of gETL NPs at the indicated temperatures. b) Schematic illustration of the experiment to characterize the antitumor effect of gETL NPs in combination with Pt thermos‐chemotherapy under the simulated working conditions of HIPEC in vitro. c) Viability of CT26 cells treated with gETL NPs, DTX‐liposomes, Pt at 37 °C. d) Viability of CT26 cells treated with gETL NPs, DTX‐liposomes, Pt at 42 °C. e) Flow cytometry analysis of the apoptosis of CT26 cells treated with gETL NPs, DTX‐liposomes, Pt at 42 °C was detection by flow cytometry. f) Quantitative analysis of apoptosis based on flow cytometry. The data was presented as the mean ± SD from three repeated experiments, One‐way ANOVA was used to determine statistical differences. (*p < 0.05, **p < 0.01, ns: not significant). Lips, DTX‐liposomes; NPs, GM‐CSF and DTX loaded gETL NPs; Pt, Oxaliplatin.

We assessed the antitumor effect of G/D‐gETL NPs under condition of HIPEC in vitro. First, we determined the drug release profile at the HIPEC relevant temperatures. Results in **Figure** **4**a showed that the releases of DTX within the first 60 min were limited to 11% and 34% at 37 and 39 °C, respectively. In contrast, 77% of DTX was released within the first 10 min at 42 °C. Next, we characterized the cytotoxicity of gETL NPs with and without combination of Pt thermo‐chemotherapy on CT26 cells (Figure 4b). We found that control NPs without drug loading, including exosomes, liposomes, and gETL NPs, did not induce significant cytotoxicity at the tested temperatures in both CT26 cells and human normal colon epithelial cells (HCoEpic) (Figure S6, Supporting Information), suggesting that gETL NPs are potentially safe for clinical application. In contrast, the cytotoxicity for drug‐loaded NPs was greater at 42 °C than that at 37 °C (Figure 4c,d). We also found that G/D‐gETL NPs exhibited significantly greater cytotoxicity than both DTX‐loaded liposomes and free DTX. IC50s (calculated based on the absolute quantify of DTX) for G/D‐gETL NPs, DTX‐loaded liposomes and free DTX at 42 °C were 4.9, 5.4, and 8.5, respectively. We found that, at both temperatures, combination with Pt further enhanced the cytotoxicity of G/D‐gETL NPs. Consistently, flow cytometry analysis found that G/D‐gETL NPs induced apoptosis in a rate significantly greater than both DTX‐loaded liposomes and free DTX, and the rate was increased when Pt treatment was combined (Figure 4e,f).

### 2.4 G/D‐gETL NPs Promote M2 to M1 Repolarization of Macrophages

**Figure 5:**
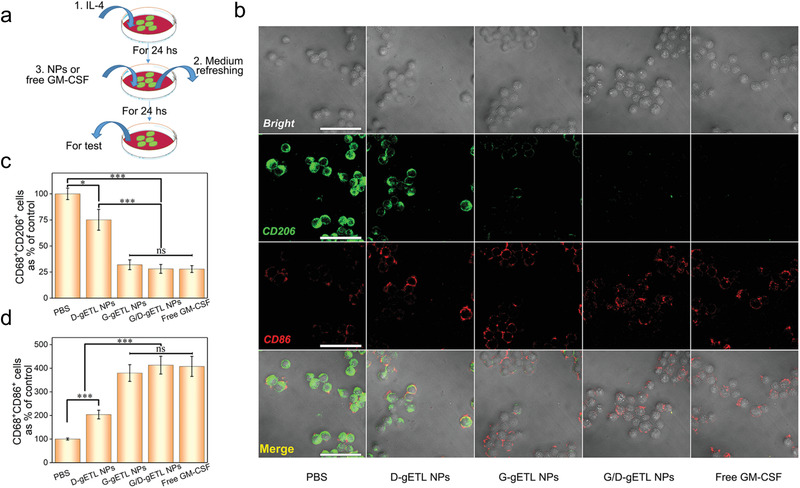
Characterization of macrophage polarization induced by gETL NPs. a) Schematic illustration of the experiment for characterization of macrophages repolarization. RAW264.7 macrophages were stimulated by IL‐4 to induce M2 phenotype, and then treated with fresh medium containing gETL NPs with various cargos or free GM‐CSF. b) Analysis of the expression of CD206 and CD86 by CLSM. Scale bar:100 µm. c) Flow cytometry analysis of the expression of M2 macrophages markers (CD68^+^ CD206^+^). d) Flow cytometry analysis of the expression of M1 macrophages markers (CD68^+^ CD86^+^). The data was presented as the mean ± SD from three repeated experiments, One‐way ANOVA was used to determine statistical differences. (*p < 0.05, **p < 0.01 ***p < 0.001, ns: not significant). D‐gETL NPs, NPs loaded with DTX; G‐gETL NPs, NPs loaded with GM‐CSF; G/D‐gETL NPs, NPs loaded with DTX and GM‐CSF.

M1 polarization is the premise of macrophages‐based antitumor immunity. G/D‐gETL NPs were loaded with GM‐CSF, an effective immune adjuvant (Figure 3e). We characterized whether treatment with G/D‐gETL NPs repolarized the M2 phenotype macrophages to M1 phenotype. RAW264.7 macrophages were induced to the M2 phenotype through treatment of IL‐4 and then incubated with G/D‐gETL NPs or free GM‐CSF (**Figure** **5**a). After 24 h, the cells were subjected to immunostaining for CD86, a M1 phenotype surface marker and CD206, a M2 phenotype surface marker, and then analyzed by CLSM. Results in Figure 5b showed that, similar to free GM‐CSF, G‐gETL, and G/D‐gETL NPs effectively converted macrophages from the M2 phenotype to the M1 phenotype. We quantified the fraction of M1 (CD68^+^CD86^+^) and M2 (CD68^+^CD206^+^) macrophages by flow cytometry (Figure S7, Supporting Information), and found that G/D‐gETL NPs converted macrophage phenotypes in efficiency comparable to free GM‐CSF, and the conversion activity was independent to the encapsulated DTX (Figure 5c,d).

### 2.5 gETL NPs Enhance Macrophage‐Mediated Tumor Cell Phagocytosis

**Figure 6:**
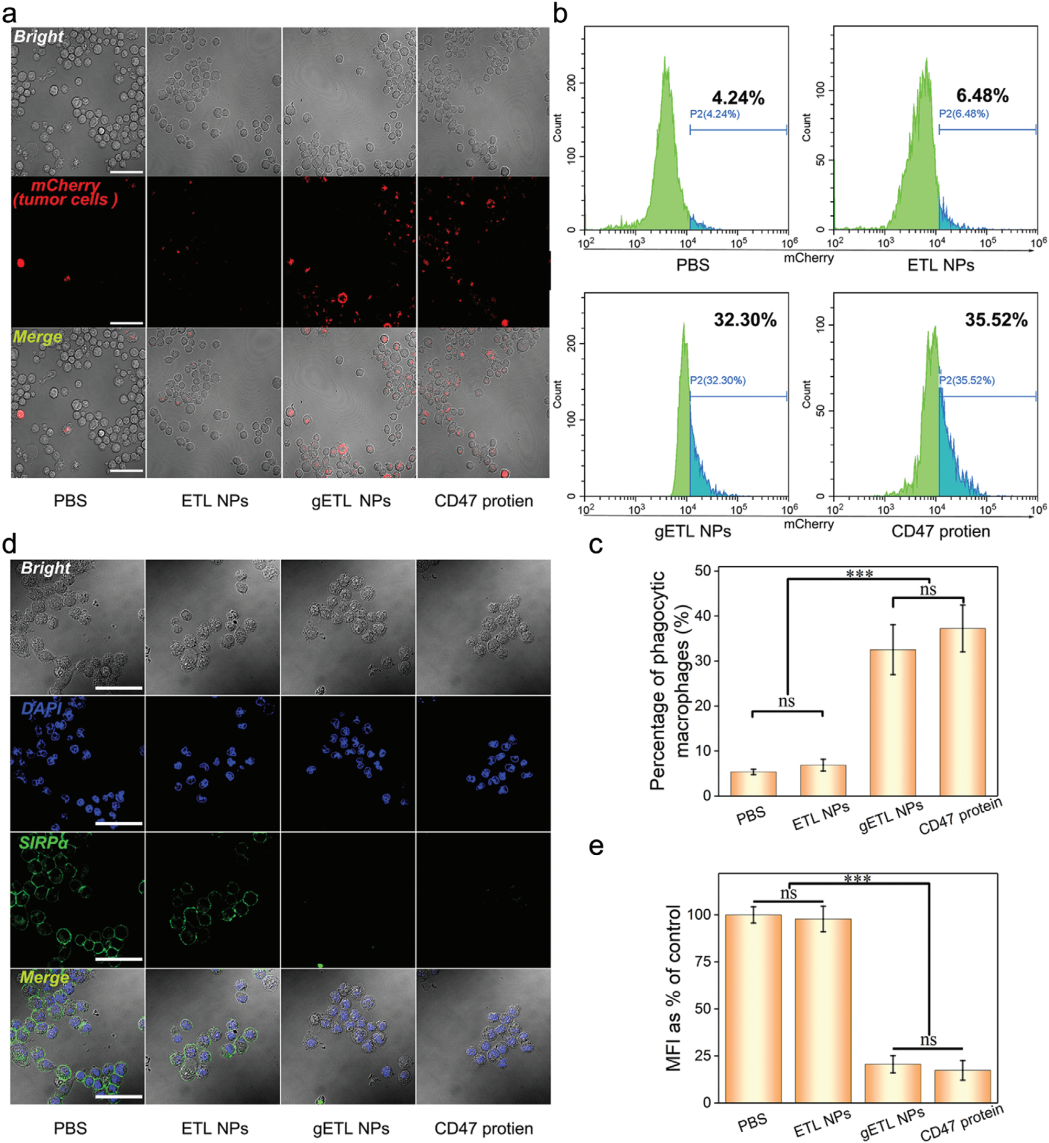
gETL NPs promoted macrophages phagocytosis of tumor cells through competitive interaction with SIRP*α*. a) CLSM analysis of phagocytosis of tumor cells by M1 macrophages. Scale bar: 100 µm. b) Flow cytometry analysis of phagocytosis of tumor cells by M1 macrophages. c) Quantification of phagocytosis of tumor cells by M1 macrophages as determined by flow cytometry. d) CLSM analysis of the expression of SIRP*α* on the surface of macrophages. Scale bar: 100 µm. e) Quantification of the mean fluorescence intensity (MFI) of SIRP*α* molecules. The data was presented as the mean ± SD from three repeated experiments, One‐way ANOVA was used to determine statistical differences. (***p < 0.001, ns: not significant).

Phagocytosis of tumor cells is a crucial step for macrophage‐mediated presentation of tumor cell antigens. gETL NPs bear CD47, a molecule which prevents phagocytosis through interaction with SIRP*α* on the surface of macrophages.^[^ ^39^, ^40^, ^41^, ^42^ ^]^ We speculated that treatment with gETL NPs would occupy SIRP*α*, thereby disengaging the interaction of SIRP*α* on macrophages with CD47 on tumor cells and thus promoting macrophage‐mediated tumor cell phagocytosis. To test the hypothesis, GM‐CSF‐induced M1 macrophages were treated with gETL NPs. Control cells were treated with ETL NPs that bear a limited amount of CD47 on the surface (Figure 3f), or free CD47 protein, which is known to enhance phagocytosis of tumor cells,^[^ ^26^ ^]^ or PBS. Macrophages after treatment were then incubated with human colon cancer HCT116 cells that were engineered to express mCherry for detection. After 6 h of incubation, phagocytosis of tumor cells was determined by CLSM (**Figure** **6**a) and flow cytometry (Figure 6b). We found that the phagocytosis efficiency in the gETL NP‐ treatment macrophages was 32.5%, which was comparable to that in CD47 protein‐treated macrophages (37.2%) and about 6 times greater than those in macrophages treated with PBS (5.4%) or ETL NPs (6.9%) (Figure 6b,c). These results suggested that the CD47 molecules displayed on the surface of gETL NPs enhance the efficiency of tumor cell phagocytosis. To validate the finding, we determined the amount of SIRP*α* molecules on the surface of macrophages after treatment through immunostaining with a FITC‐SIRP*α* antibody. CLSM analysis found that, unlike the macrophages treated with PBS or ETL NPs, which showed strong FITC fluorescence on the surface, the macrophages treated with gETL NPs or CD47 protein bear negligible fluorescence (Figure 6d). Semiquantification of FITC fluorescence found that the relative mean fluorescence intensity (MFI) values in gETL NPs‐ treated and CD47 protein‐treated macrophages were 20.6% and 17.4%, respectively; in contrast, the values for macrophages treated with PBS or ETL NPs were 97.8% (Figure 6e). Collectively, these results suggest that treatment with gETL NPs promoted phagocytosis through competitive interaction with SIRP*α* on the surface of macrophages.

### 2.6 Characterization of Biodistribution and Pharmacokinetics of gETL NPs

**Figure 7:**
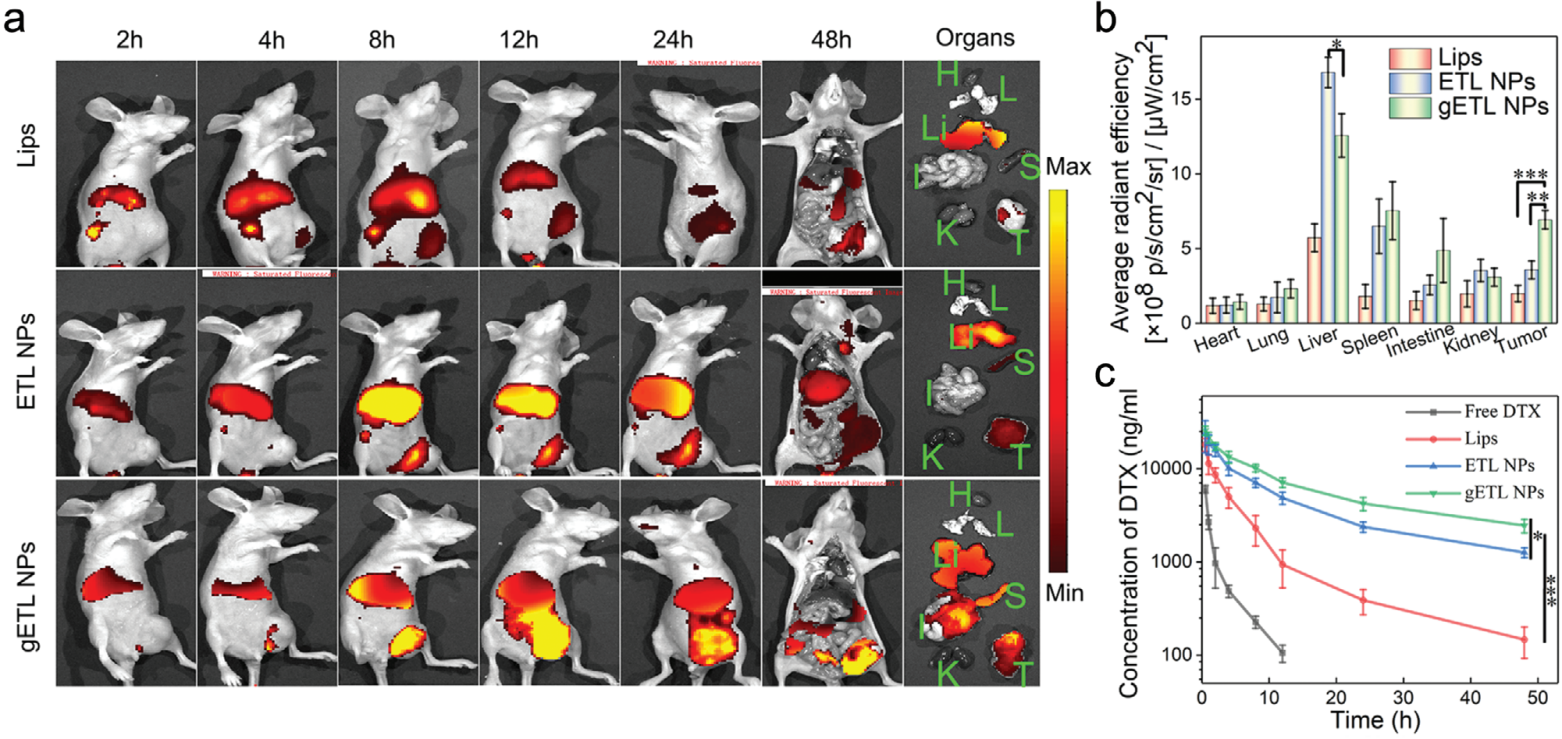
Biodistribution and pharmacokinetics of NPs in vivo. a) Representative images of the indicated NPs in mice and isolated organs. Tumor‐bearing mice were treated with the tested NPs at a DIR‐equivalent dose of 2.5 mg kg^−1^ via tail vein. After 48 h, the mice were imaged by IVIS. b) Quantification of the accumulation of NPs in major organs and tumors at 48 h postinjection based on DIR fluorescence (*n* = 3). c) Quantification of the concentration of DTX in plasma 48 h after intravenous injection of free DTX, DTX‐ liposomes, DTX‐ETL NPs, or DTX‐gETL NPs (at a DTX dose of 5 mg kg^−1^) into BABL/C mice (*n* = 3). The data was presented as the mean ± SD from three repeated experiments, One‐way ANOVA was used to determine statistical differences. (*p < 0.05, **p < 0.01, ***p < 0.001). Abbreviation: H, heart; L, lung; Li, liver; S, spleen; I, intestines; K, kidney; T, tumor.

We determined the biodistribution of gETL NPs after intravenous administration. A patient derived xenograft (PDX) mouse model of mPC, which was established through transplantation of mPC tumors isolated from a human patient into the peritoneal cavity of BALB/c nude mice according to published procedures,^[^ ^43^ ^]^ was used. To enable in vivo imaging, tested NPs, including gETL NPs, ETL NPs and liposomes, were synthesized with encapsulation of DiR, a fluorescence dye. When tumor sizes reached 50 mm^3^, the mice were treated with the selected NPs at a DiR‐equivalent dose of 2.5 mg kg^−1^ through intravenous administration. At 2, 4, 8, 12, 24, and 48 h time points, the mice were imaged using an in vivo imaging system (IVIS). We found that the accumulation of liposomes in the liver was significantly greater than that in tumors and most of liposomes were eliminated by 48 h (**Figure** **7**a). A similar distribution pattern was found in mice received intravenous administration of ETL NPs, although the overall fluorescence intensity was stronger than that in the liposome group. In contrast, the accumulation of gETL NPs in tumors, which peaked at 12 h postadministration, was significantly greater than that in mice received ETL NPs across all the time points. Semiquantitation based on fluorescence intensity showed that the accumulation of gETL NPs in tumors was 3.3 and 2.1 times greater than those in liposome‐ treated and in ETL NP‐treated mice at 48 h postinjection, respectively (Figure 7b). The high accumulation of gETL NPs in the liver at the 48 h time point could be attributed to their long blood circulation time (Figure 7c). Then we further determined the blood circulation of NPs that were synthesized with encapsulation of DTX. Mice treated with free DTX, DTX‐loaded liposomes, or DTX‐loaded ETL NPs were included as controls. We found that, compared to the controls, gETL NPs had significantly prolonged blood circulation time. The clearance half times (*t*_1/2*β*_) for DTX, liposomes, ETL NPs and gETL NPs were 0.5, 3.5, 6.2, and 8.1 h, respectively (Figure 7c). The prolonged in vivo circulation observed in the ETL NPs group compared to the Lips group could be attributed to the expression 5% of CD47 (Figure 3f). Taken together, those results suggest that surface display of CD47 prolongs the blood circulation of NPs and enhances the accumulation of NPs in tumors.

### 2.7 Characterization of G/D‐gETL NPs for mPC Treatment in CT26‐Derived mPC Xenografts

**Figure 8:**
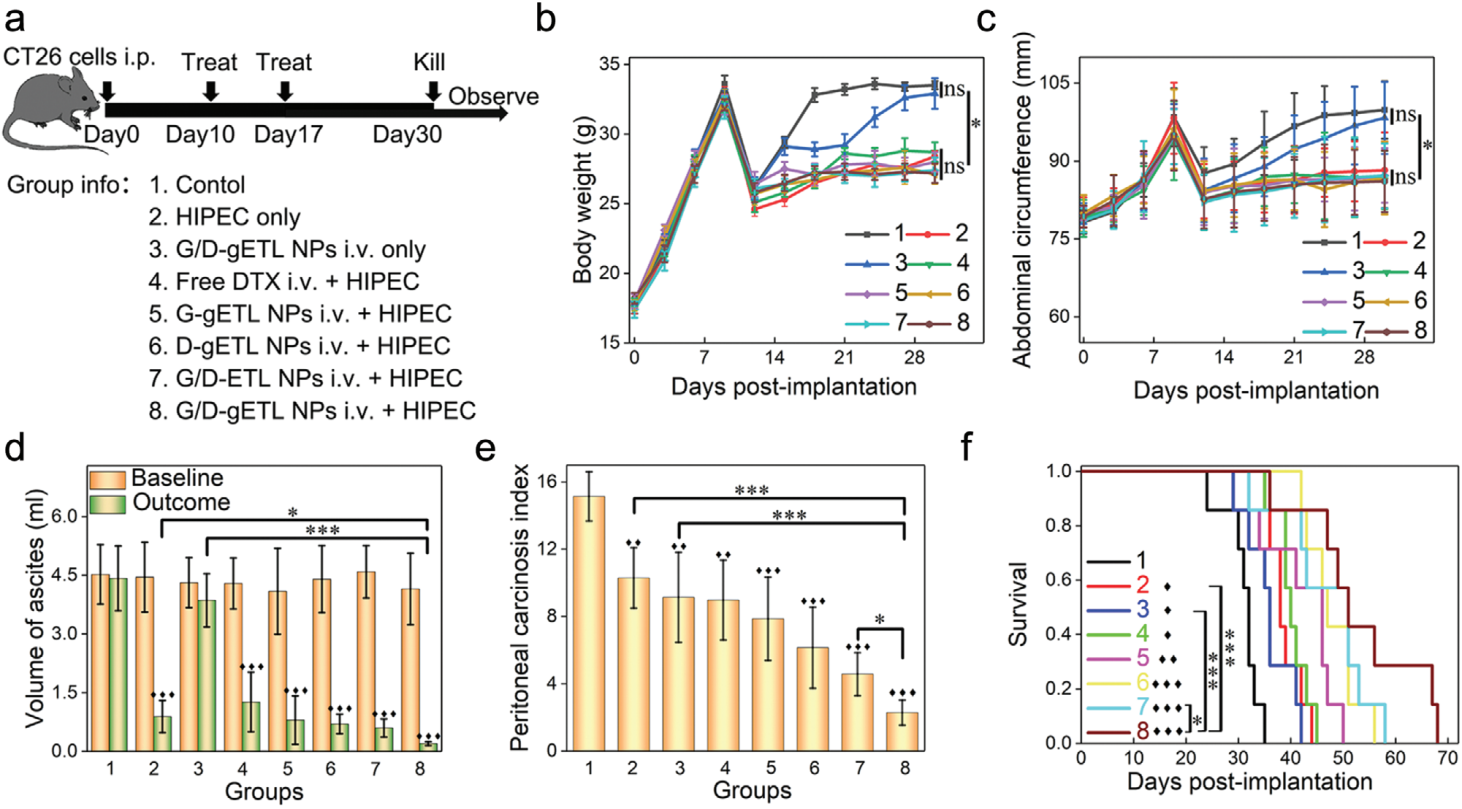
Characterization of the therapeutic benefits of NP treatment in CT26‐derived mPC xenografts. a) Schematic illustration of the experimental design. b) Change of body weight with time. c) Change of abdominal circumference with time. d) Ascites baseline and outcome of animals. e) Outcome of tumor burden for animals in term of peritoneal carcinosis index (PCI). f) Kaplan‐Meier survival analysis of mice received the indicated treatment. Median survival time was used for statistical analysis. The data was presented as the mean ± SD, One‐way ANOVA was used to determine statistical differences (♦, *p* < 0.05, ♦♦, *p* < 0.01, ♦♦♦, *p* < 0.001, compared with control group; *p < 0.05, **p < 0.01, ***p < 0.001, ns: not significant, compared with the indicated groups).

We assessed the antitumor effect of G/D‐gETL NPs in combination with HIPEC in CT26‐derived mouse model of mPC. Ten days after tumor cell inoculation, mice were randomly grouped (*n* = 12 per group) and received treatments of free drugs or drug‐loaded NPs as shown in **Figure** **8**a. The treatments were administered intravenously after ascites were removed by abdominal puncture. For those groups involved with HIPEC treatment, Pt‐based HIPEC was performed 12 h after the initial treatment (Figure 8a). On day 17, the mice received the second cycle of treatment according to the same procedures but without removal of ascites. The mice were then monitored for changes of body weight and abdominal circumference every 3 days. By day 30, 5 mice from each cohort were euthanized. Blood samples were collected for biochemical analysis. Ascites was collected and measured. Tumor burden was evaluated using the experimental peritoneal carcinomatosis index (PCI) system. The rest of mice were continuously monitored for survival (Figure 8a). We found that the abdominal circumference and body weight of mice in each group gradually increased with time and peaked at day 10. We set the values collected at this time point as the baselines (Figure S8, Supporting Information). After two cycles of treatment, the mice in all the HIPEC‐treated groups showed stable body weights (Figure 8b) and abdominal circumference (Figure 8c). The mice in the HIPEC‐treated groups produced significantly less ascites and the group treated with G/D‐gETL NPs + HIPEC had the least volume. In contrast, the volume of ascites in the group received treatment of G/D‐gETL NPs without HIPEC remained at a high level (Figure 8d; Figure S8, Supporting Information). Consistently, PCI analysis found that G/D‐gETL NPs + HIPEC treatment led to the greatest tumor inhibition rate (85.1% reduced PCI of control), following with combination of GM‐CSF‐ or DTX‐loaded gETL NPs, or G/D‐ETL NPs, and HIPEC; while other treatment options resulted in significant less inhibition effect, and the inhibition rate in the G/D‐gETL NPs + HIPEC treatment group was 2.6 times greater that HIPEC alone (Figure 8e). These data suggest that HIPEC is effective on controlling ascites, and this effect could not be achieved through intravenous chemotherapy (Figure 8d). Compared with the combination of free DTX and HIPEC, the combination of DTX‐loaded gETL NPs with HIPEC showed significantly greater efficacy, suggesting that gETL NPs as a delivery vehicle provide significant therapeutic benefit. We found that, while encapsulation of GM‐CSF or DTX alone in NPs provided therapeutic benefit beyond HIPEC, the formulation with encapsulation of both agents produced significantly greater efficacy (Figure 8d,e). In the histological analysis, we characterized the apoptosis of tumor cells in residual tumors. CLSM of TUNEL staining identified significant apoptosis in tumors in these groups received the combination of NPs with HIPEC (Figure S9, Supporting Information). Quantitative analysis showed that the apoptotic rates in those groups treated with either HIPEC or NPs alone were limited to 6%, while the rates in the groups treatment with combinatorial regimens reach up to 25–58% (Figure S10, Supporting Information). The median survival for mice treated with G/D‐gETL NPs + HIPEC reached 53 days, which was 5–15 days longer than those for control treatment or those for groups received treatment of single regimen (Figure 8f). We found that the additional therapeutic benefit from the use of NPs was not because of their systemic toxicity, as hematological analysis of serum biochemical indicators (Figure S11, Supporting Information) and histological analysis of major organs (Figure S12, Supporting Information) did not identify systemic toxicity.

**Figure 9:**
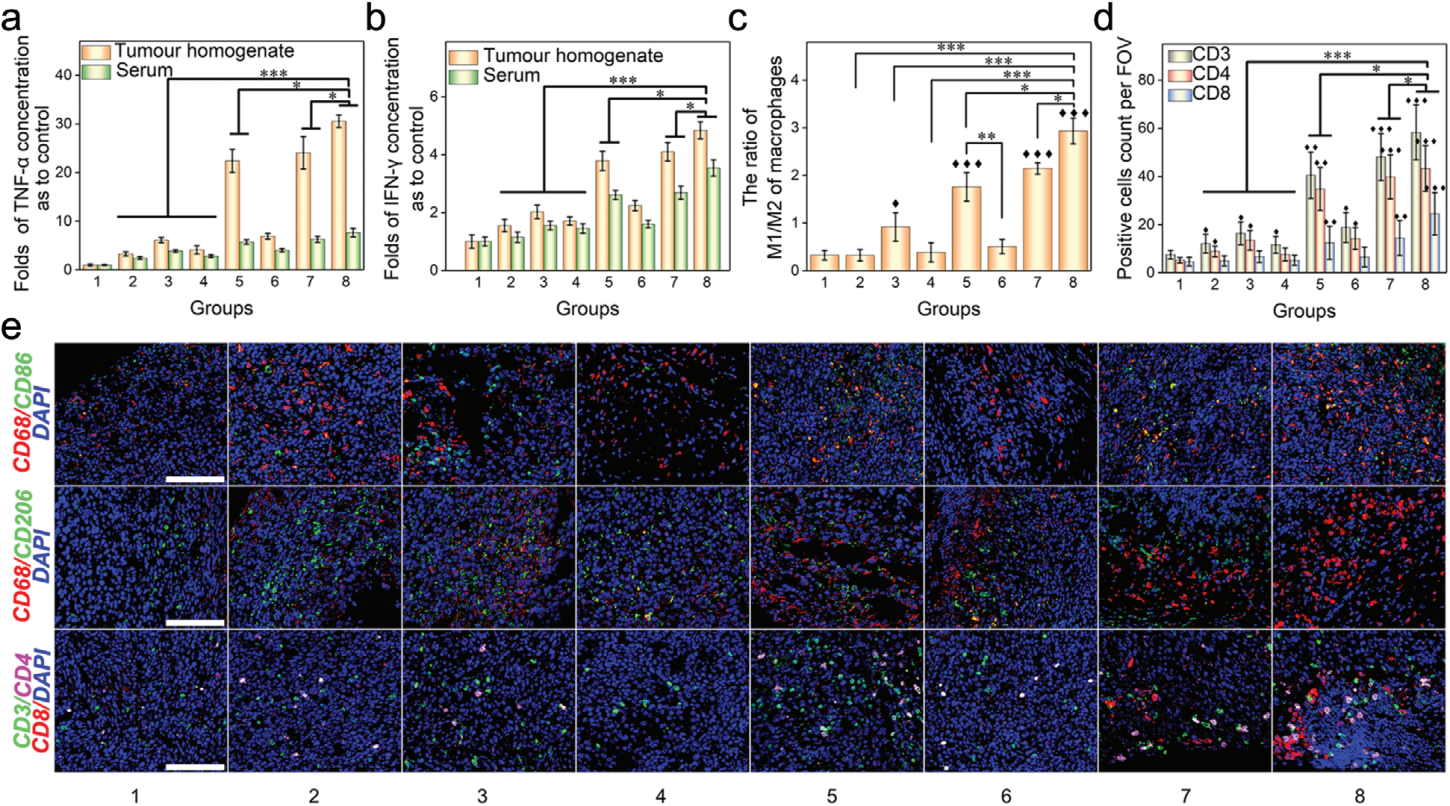
Characterization of the antitumor mechanism. a) Fold‐changes of TNF‐*α* concentration in tumor homogenate and serum compared with control group. b) Fold‐changes of IFN‐*γ* concentration in tumor tissue homogenate and serum compared with control group. c) Quantitative analysis of the ratio of M1 macrophages (CD86^+^)/M2 macrophages (CD206^+^) by immunofluorescence staining of tumor sections. d) Quantitative analysis of tumor infiltrating T cells (CD3^+^), CD4 T cells (CD3^+^ CD4^+^), and CD8 T cells (CD3^+^CD8^+^) of mPC tumors. Mean count of positive cells from 5 high‐power field per section was used for statistical difference analysis, One‐way ANOVA was used to determine statistical differences; ♦, *p* < 0.05, ♦♦, *p* < 0.01, ♦♦♦, *p* < 0.001, compared with control group; *p < 0.05; **p < 0.01, ***p < 0.001, compared with the indicated groups. e) Immunofluorescence staining of different macrophages markers (CD68^+^ CD86^+^ or CD68^+^ CD206^+^) and T cells markers (CD3^+^, CD4^+^, CD8^+^) in tumor tissue sections. Scale bar: 100 µm.

We investigated the mechanisms accounting for the observed antitumor effects. First, we assessed if the treatments activated systemic immune response by measuring the changes of proinflammatory cytokines TNF‐*α* and IFN‐*γ* in the blood samples and tumors.^[^ ^44^ ^]^ Results in **Figure** **9**a,b showed that treatment with G/D‐gETL NPs + HIPEC induced the highest level of cytokines. The level of TNF‐*α* in this group was 4–9 times greater than those in mice treated with gETL NPs without GM‐CSF and those treatments without NPs, and the level of difference for IFN‐*γ* is 2–3 times. These results suggested that antitumor immunity was activated by the encapsulated GM‐CSF, whose activity was improved when HIPEC is combined. Next, we characterized the effects of various treatments on macrophage polarization. Immunofluorescence analysis found that the ratios of M1 macrophages (CD68^+^ CD86^+^) to M2 macrophages (CD68^+^ CD206^+^) in mice treated with GM‐CSF loaded NPs were significantly higher than those in mice received treatment without GM‐CSF, and the group treated with G/D‐gETL NPs + HIPEC had the highest ratio of 2.9, following with GM‐CSF‐loaded gETL NPs + HIPEC (2.1) and GM‐CSF/DTX‐loaded ETL NPs + HIPEC (1.8) (Figure 9c,e). The data suggested that the encapsulated GM‐CSF regulated macrophages polarization and the polarization effect was enhanced when HIPEC was combined. Last, as it is known that macrophages after activation can recruit and activate T cells, which in turn further enhance the antitumor immune response,^[^ ^45^ ^]^ we examined that treatments changed the infiltration of CD3^+^ CD4^+^ and CD3^+^ CD8^+^ T‐cells in tumors. We found that those groups treated with GM‐CSF‐loaded NPs in combination with HIPEC presented significantly larger amounts of T cells than other groups, with the group treated with G/D‐gETL NPs + HIPEC having the greatest amount of T cells (Figure 9d,e). The infiltration of CD8^+^ T cells in tumor microenvironment was determined by GM‐CSF mediated macrophages polarization and tumor antigen exposure in tumor microenvironment. Mice in both group 5 and group 8 were treated with gETL NPs. On top of this, mice in group 8 were co‐treated with DTX. As a chemotherapy drug, DTX can induce apoptosis in tumors, which subsequently attracts more T cells. Therefore, compared to group 8, mice in group 5 have lower CD8^+^ T cells in tumors. Different from group 8, group 7 were treated with ETL NPs. Due to the lower expression of CD47, the tumor accumulation of NPs in group 7 was less than those in group 8. As a result, compared with group 8, there was less M1 macrophage polarization and thus lower CD8^+^ T cells infiltration in group 7 (Figure 9d,e).

### 2.8 Validation of the Antitumor Effects in a PDX Model

**Figure 10:**
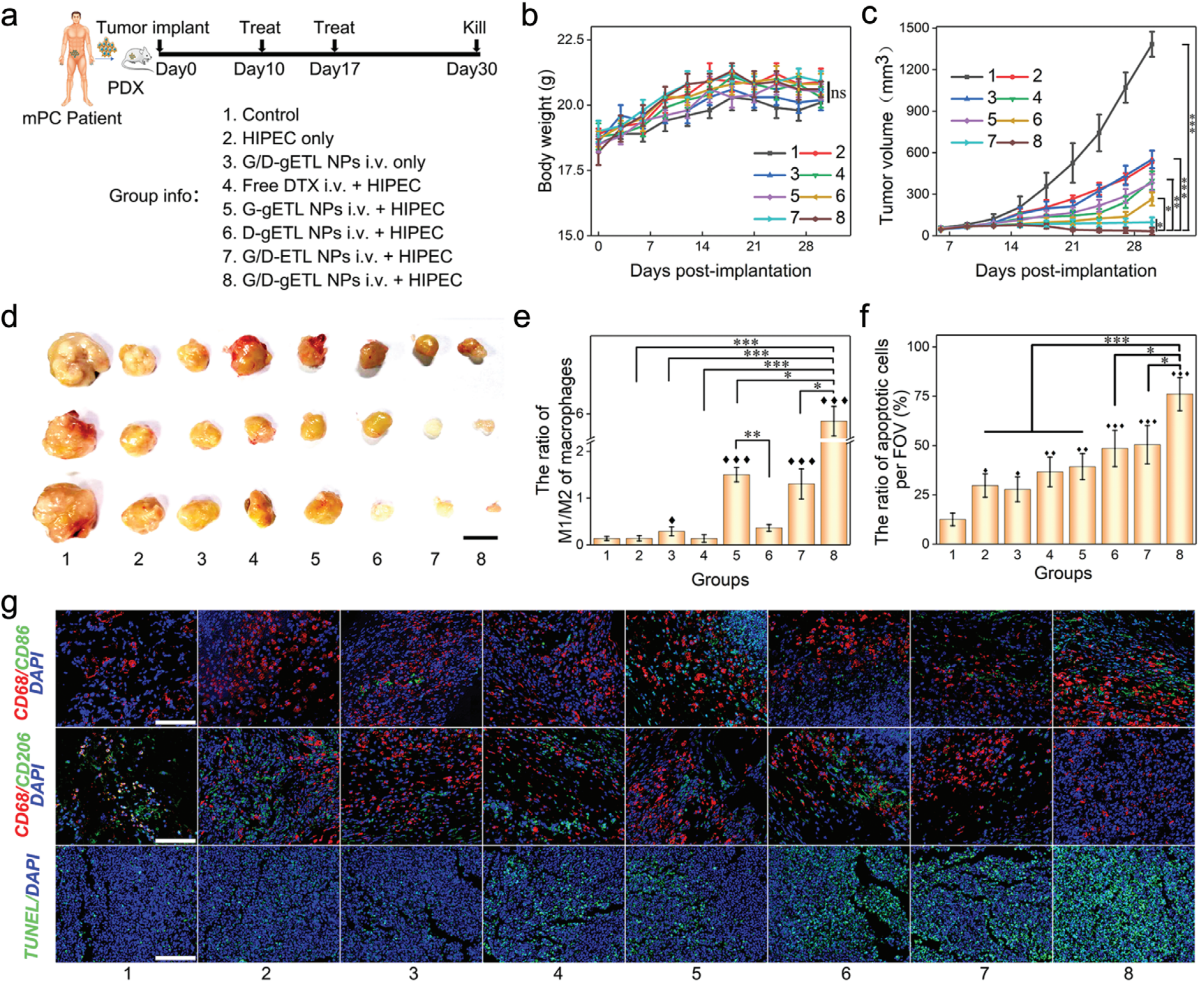
Characterization of the therapeutic benefits of NPs treatment in patient‐derived mPC tumor xenografts (*n* = 3). a) Schematic illustration of the experimental design. b) Change of body weight with time. c) Change of body weight with time. d) Outcome of tumor volume at the observing endpoint (day 30), scale bar: 1 cm. e) Quantitative analysis of the ratio of M1 macrophages (CD86^+^)/M2 macrophages (CD206^+^) by immunofluorescence staining of tumor sections. f) Quantification of apoptosis of tumor cells detected by TUNEL staining. g) Analysis of tumors by TUNEL staining and immunofluorescence staining of the indicated macrophages markers (CD68^+^, CD86^+^, and CD206^+^) in tumor sections post‐treatments, bar: 100 µm. Mean count (e) or mean apoptotic rate (c) of positive cells from 5 high‐power field per section was used for statistical difference analysis, One‐way ANOVA was used to determine statistical differences; ♦, *p* < 0.05, ♦♦, *p* < 0.01, ♦♦♦, *p* < 0.001, compared with control group; *, *p* < 0.05; **, *p* < 0.01, ***, *p* < 0.001, compared with the indicated groups.

Due to the unique pathophysiology, human mPC may not be fully recapitulated in cell‐line‐derived xenografts (CDX).^[^ ^46^ ^]^ To validate the therapeutic benefit found in the CT26‐ derived CDX model, we evaluated the antitumor effects of various treatments in the PDX model that was utilized in the aforementioned biodistribution study. Therapeutic evaluation was carried out according to the procedures described in **Figure** **10**a. We found that the overall therapeutic benefits of various treatments were similar to those found in the CT26‐derived CDX model, except that no significant differences in body weight and ascites were observed among various groups (Figure 10b; Figure S13, Supporting Information). We found that tumors in mice treated with HIPEC alone or HIPEC in combination with free DTX were inhibited initially but re‐grew with time. By the end of the study, treatment with G/D‐gETL NPs or HIPEC alone resulted in 60% and 58% tumor inhibition, respectively (Figure 10c,d). In contrast, treatment with GM‐CSF‐loaded gETL NPs + HIPEC and DTX‐loaded gETL NPs + HIPEC achieved 71% and 81% tumor inhibition, respectively. We found that display of CD47 on NPs was beneficial, as G/D‐gETL NPs + HIPEC achieved remarkable 97% inhibition, compared to 90% by G/D‐ETL NPs + HIPEC. The inhibition rate by G/D‐gETL NPs + HIPEC treatment was 1.7 times greater than treatment with HIPEC alone (Figure 10d). Similar to the findings in the CDX study, GM‐CSF loaded NPs induced significant M1 polarization of macrophages, and combination with HIPEC augmented the polarization effect (Figure 10g). For instance, compared with those groups without GM‐CSF, treatment with GM‐CSF‐loaded NPs increased the number of CD86^+^ cells while decreased the number of CD206^+^ cells (Figure 10e). The groups treated with GM‐CSF showed significant higher ratio of M1 to M2 compared to the groups without GM‐CSF. Among them, the G/D‐gETL NPs + HIPEC demonstrated a significantly high M1 to M2 ratio at 5.5. TUNEL staining found that treatment with G/D‐gETL NPs + HIPEC induced the highest apoptotic rate of 76.1%, following with DTX‐loaded gETL NPs + HIPEC (50.4%), and GM‐CSF‐loaded gETL NPs + HIPEC (48.5%). The apoptotic rates in mice treated with HIPEC or NPs alone were below 30% (Figure 10f). Additionally, tumor sections were staining for HE, HER2, and Ki‐67 and compared with patient‐derived tumors to confirm the homogeneity of the PDX tumor model (Figure S14, Supporting Information).

## 3 Discussion

The therapeutic benefit of HIPEC has been limited by various factors, such as inefficient drug penetration and resistance to standard chemotherapy drugs.^[^ ^8^, ^10^, ^14^, ^47^ ^]^ In this study, we quantified the drug delivery efficiency of HIPEC at the cellular level using single‐cell ICP‐MS, and found that, compared with small sized tumors, larger tumors are associated with limited drug penetration (Figure 2). To improve drug delivery to tumors, we developed a novel delivery system through fusion of genetically engineered exosomes and thermosensitive liposomes. We demonstrated that the resulting gETL NPs after intravenous administration accumulate preferentially in tumors, efficiently release payloads at the temperature utilized in HIPEC therapy, (Figure 4a), making them ideal for targeted drug delivery preferentially to tumors for combination therapy with HIPEC. gETL NPs bear CD47, a molecule known to facilitate cargos to escape from phagocytosis,^[^ ^22^, ^48^, ^49^ ^]^ and, as a result, are capable of circulating in the blood over long time, leading to enhanced accumulation in tumors (Figure 7). In addition, the display of CD47 molecule enhances macrophage‐mediated tumor cell phagocytosis. We explored treatment of mPC through combination of local HIPEC and systemic administration of G/D‐gETL NPs in both CDX and PDX models. We found that, compared to either regimen alone, the combination provided significantly greater therapeutic benefits, which are likely resulted from multiple lines of antitumor mechanisms, including DTX‐ and HIPEC‐mediate chemotherapy and CD47‐ and GM‐CSF‐mediated immunotherapy.

## 4 Conclusions

In conclusion, we demonstrated that drug delivery in the standard HIPEC therapy is insufficient and limited by the size of tumor nodules. To improve drug delivery to mPC, we developed a novel gETL NP‐based delivery system and demonstrated that the NPs could be utilized for delivery of therapeutic agents to improve mPC treatment. This study suggests a new direction to enhance treatment of mPC through combination of HIPEC with gETL NP‐mediated chemotherapy and immunotherapy.

## Conflict of Interest

The authors declare no conflict of interest.

## Supporting information

Supporting Information
