# Supplementary material for: Thermosensitive Exosome–Liposome Hybrid Nanoparticle‐Mediated Chemoimmunotherapy for Improved Treatment of Metastatic Peritoneal Cancer
Source: Adv Sci (Weinh). 2020 Jul 29;7(18):2000515. doi: 10.1002/advs.202000515 (PMC7509655; doi:10.1002/advs.202000515)
Supplement: Supplementary file 1 — Supporting Information [file ADVS-7-2000515-s001.pdf]

Copyright WILEY-VCH Verlag GmbH & Co. KGaA, 69469 Weinheim, Germany, 2020.

ADVANCED SCIENCE
Open Access

Supporting Information

for *Adv. Sci.*, DOI: 10.1002/advs.202000515

Thermosensitive Exosome–Liposome Hybrid Nanoparticle-Mediated Chemoimmunotherapy for Improved Treatment of Metastatic Peritoneal Cancer

Qijun Lv, Lili Cheng, Yao Lu, Xiaoge Zhang, Yizhen Wang, Junfeng Deng, Jiangbing Zhou, Bo Liu,* and Jie Liu**

Supporting Information including additional Results and Methods for Article:

Thermosensitive Exosome-liposome Hybrid Nanoparticle-mediated Chemoimmunotherapy for Improved Treatment of Metastatic Peritoneal Cancer

Qijun Lv, Lili Cheng, Yao Lu, Xiaoge Zhang, Yizhen Wang, Junfeng Deng, Jiangbing Zhou, Bo Liu*, and Jie Liu**

Q. Lv, Y. Wang, J. Deng, and Prof. B. Liu

Department of General Surgery, the Third Affiliated Hospital of Sun Yat-sen University

Guangzhou 510120, China

L. Cheng, Y. Lu, X. Zhang, and Prof. J. Liu

School of Biomedical Engineering, Sun Yat-sen University

Guangzhou, Guangdong 510006, China

Prof. J. Zhou

Departments of Neurosurgery and Biomedical Engineering, Yale University

New Haven, CT 06511, USA

* Corresponding Author:

Email: liujie56@mail.sysu.edu.cn; jiangbing.zhou@yale.edu.cn; liubo3@mail.sysu.edu.cn

Results

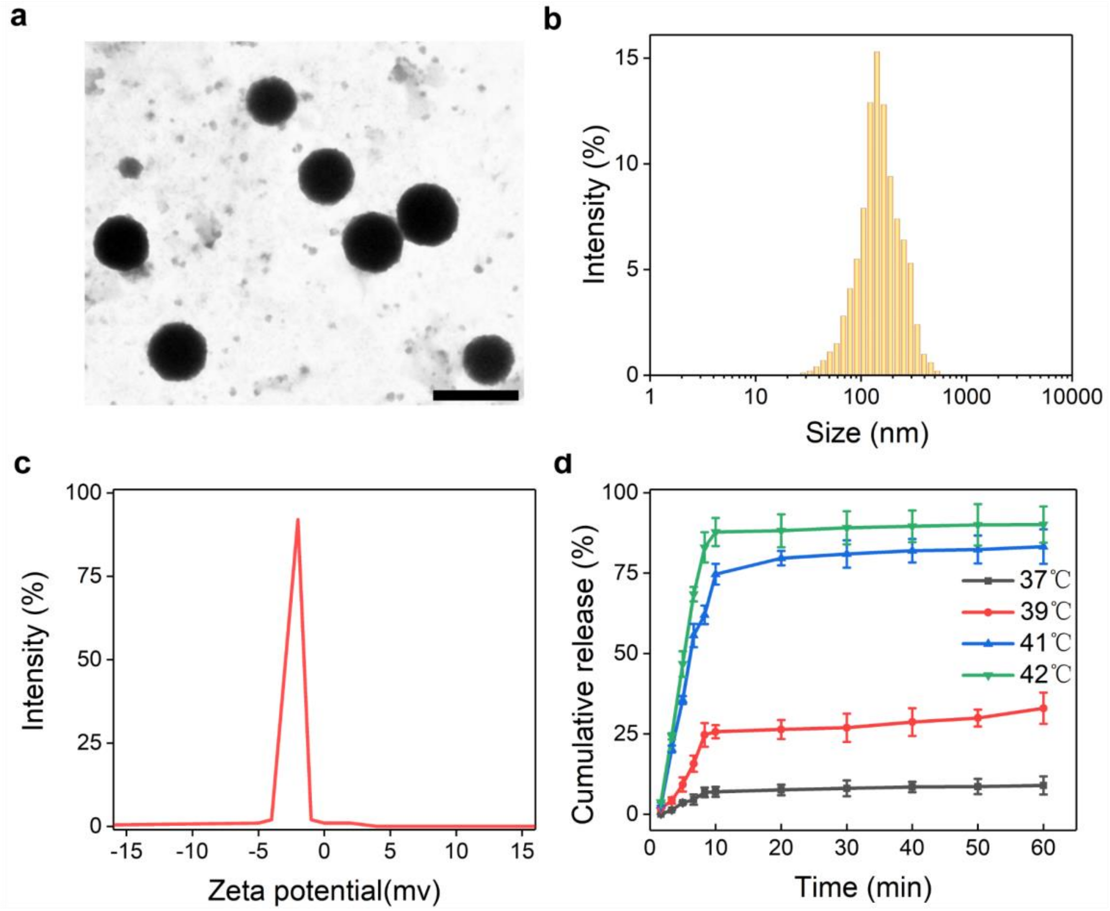

Figure S1. Characterization of thermosensitive liposomes. a) The morphology of liposomes was detected by TEM, scale bar: 200 nm. **b)** Size distribution of liposomes. **c)** Zeta potential of liposomes. **d)** Release profiles of DTX from liposomes in 10% serum PBS solution (10 mM, pH 7.4) at different temperatures. The data was presented as the mean ± SD from three repeated experiments.

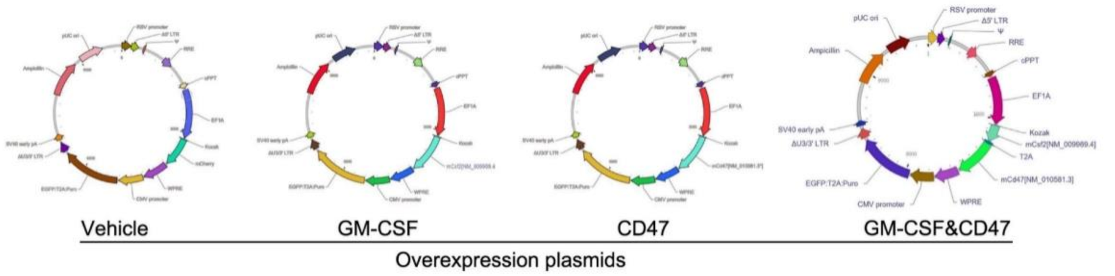

Figure S2. Schematic illustration of constructed strategies of overexpression plasmids

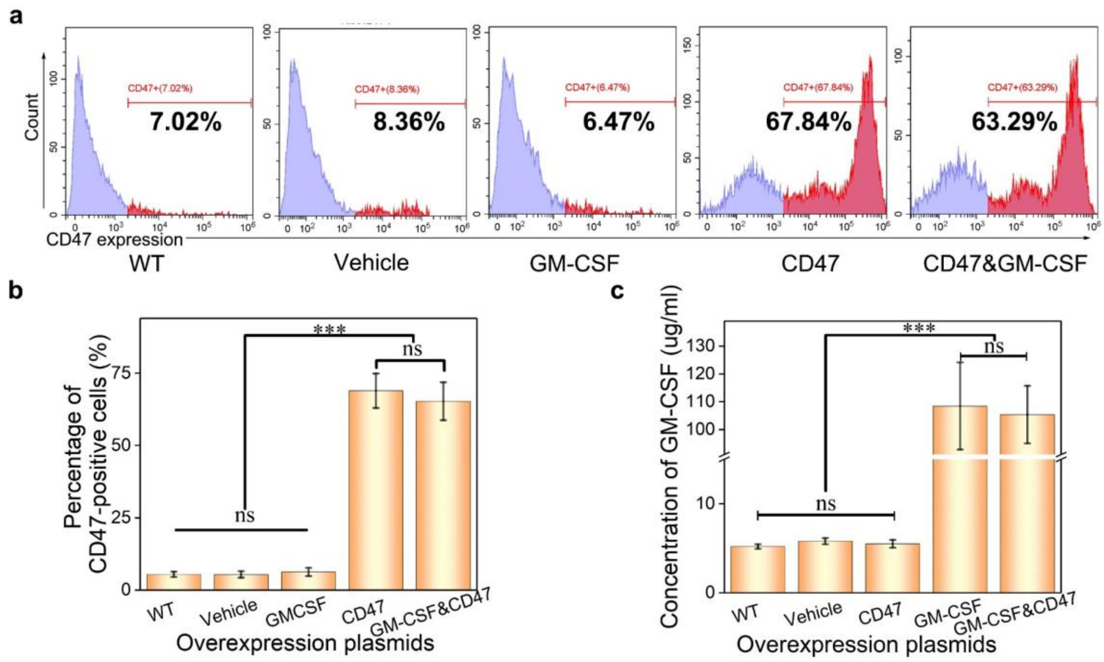

Figure S3. CD47 and/or GM-CSF overexpressed cells were constructed by lentivirus transfection. a) Flow cytometry analysis of CD47 overexpression in cells transfected with different plasmids, cells collected from each group were stained with FITC-CD47 antibody and detected by flow cytometry. **b)** Quantitative analysis of CD47 expression based on data of flow cytometry analysis. **c)** Expression levels of GM-CSF in each group were detected by ELISA. The data was presented as the mean ± SD from three repeated experiments, One-way ANOVA was used to determine statistical differences. (* $p < 0.05$, *** $p < 0.001$, ns: not significant).

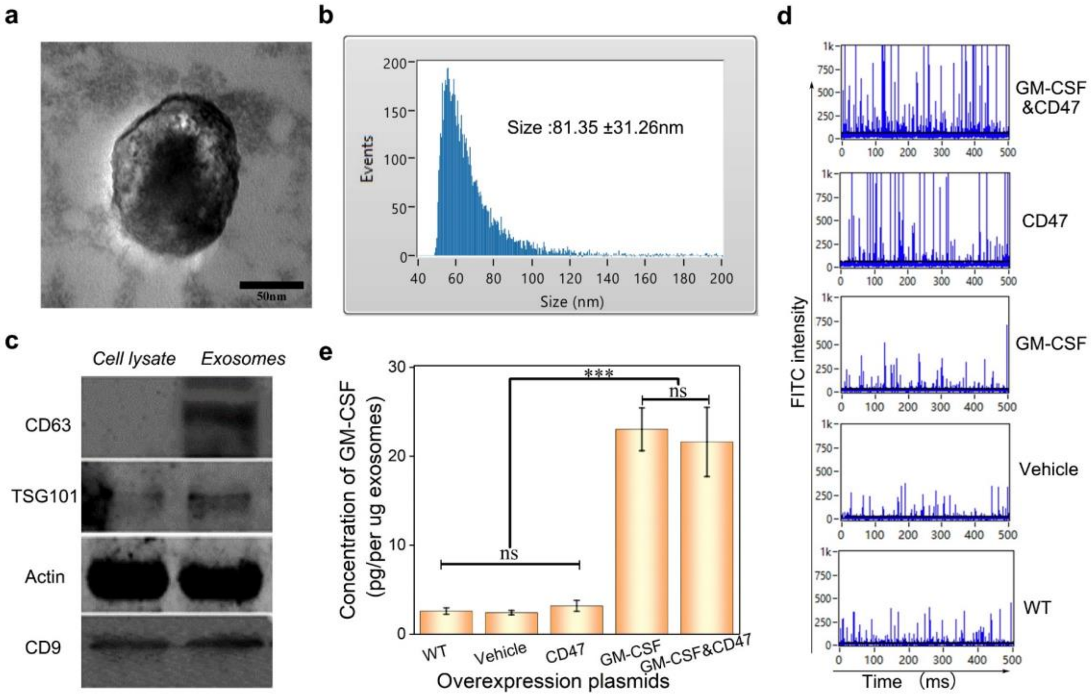

Figure S4. Characterization of genetically engineered exosomes. a) The morphology of exosome was detected by TEM, scale bar: 50 nm. **b)** The size distribution of exosomes was measured by Flow NanoAnalyzer. **c)** Western blot analysis markers of exosomes including CD63, TSG101, CD9. **d)** The expression level of CD47 in exosomes derived from different plasmid transfected cell lines was measured, exosomes derived from each group were stained with FITC-CD47 antibody and detected by Flow NanoAnalyzer. Each pulse represented the intensity of CD47 expression on a single vesicle. **e)** The amount of GM-CSF in exosomes derived from mouse fibroblasts was detected by ELISA. Three independent experiments were repeated. WT, wild type.

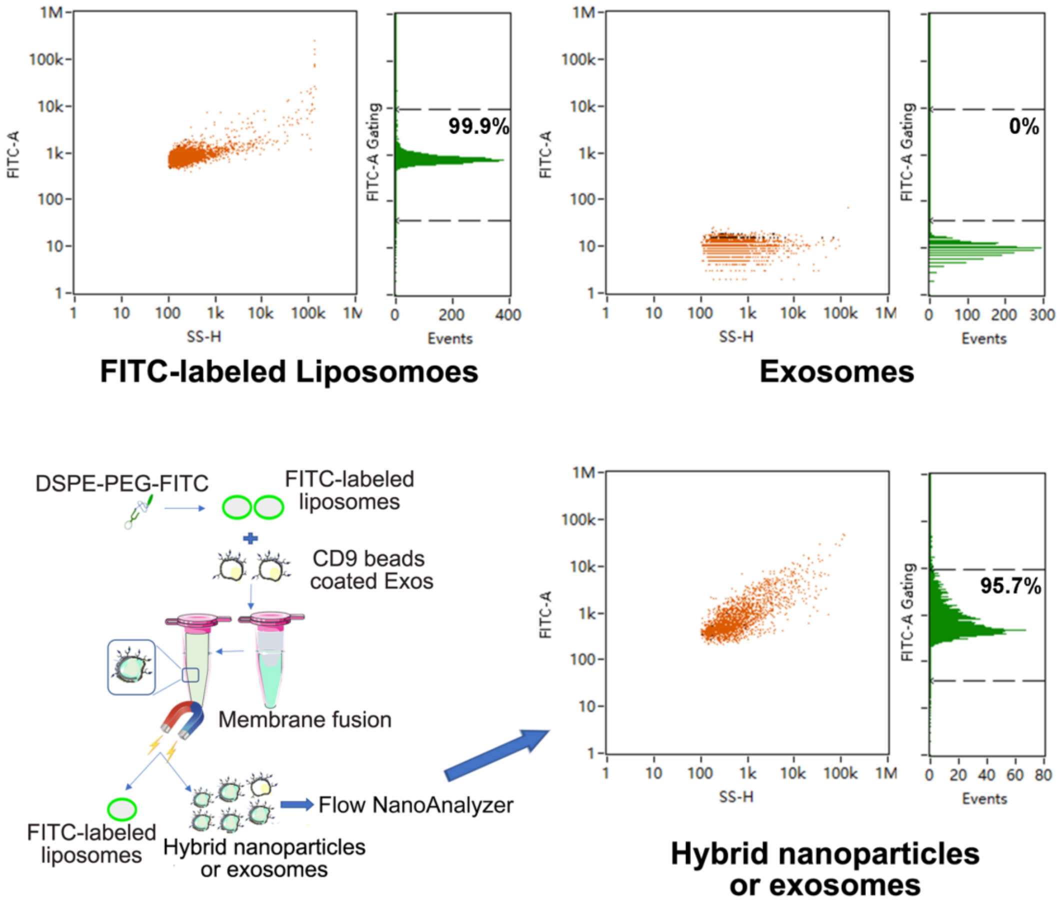

Figure S5. Quantitative analysis of fusion efficiency of liposomes and exosomes. The experiments were carried out in triplicate.

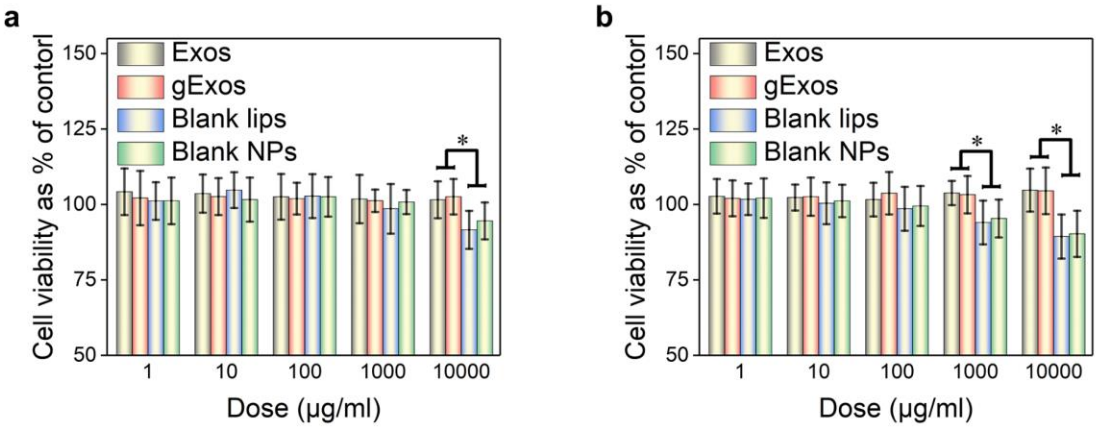

Figure S6. Characterization of cytotoxicity of the indicated nanoparticles CT26 cells (a) or HCoEpic cells (b). Exos, wild type exosomes; gExos, exosomes with CD47&GM-CSF overexpression; Blank lips, blank thermosensitive liposomes; Blank NPs, engineered exosomes hybridized with blank liposomes. The data was presented as the mean ± SD from three repeated experiments, One-way ANOVA was used to determine statistical differences. (* $p < 0.05$).

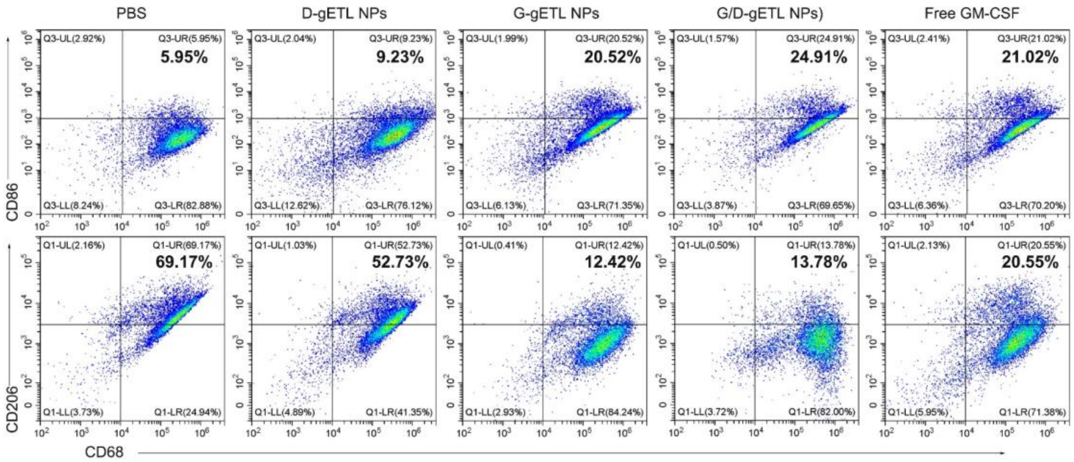

Figure S7. M2 to M1 repolarization of RAW264.7 macrophages induced by gETL NPs was evaluated by flow cytometry. The representative percentages of M1 macrophages ($CD68^+$ $CD86^+$) and M2 macrophages ($CD68^+$ $CD206^+$) were displayed here, the quantification results were analyzed and showed in **Figure 5c and 5d**, three independent experiments were repeated.

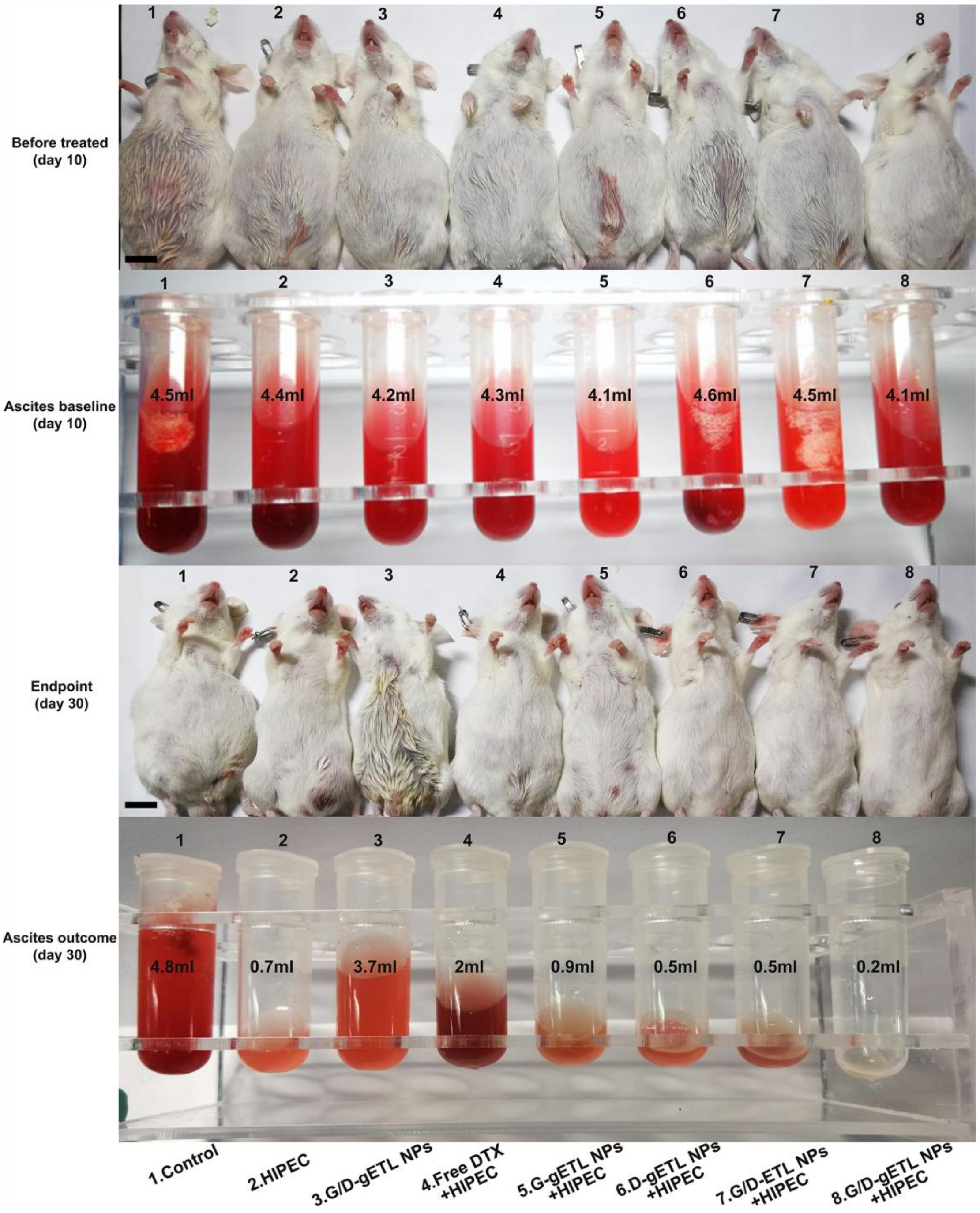

Figure S8. Synergistic therapeutic benefits of G/D-gETL NPs and HIPEC in CT26-derived mPC xenografts (n=5). Abdominal circumference and ascites at pre-treatment (day 10) and at endpoint (day 30) were recorded, scale bar: 1 cm.

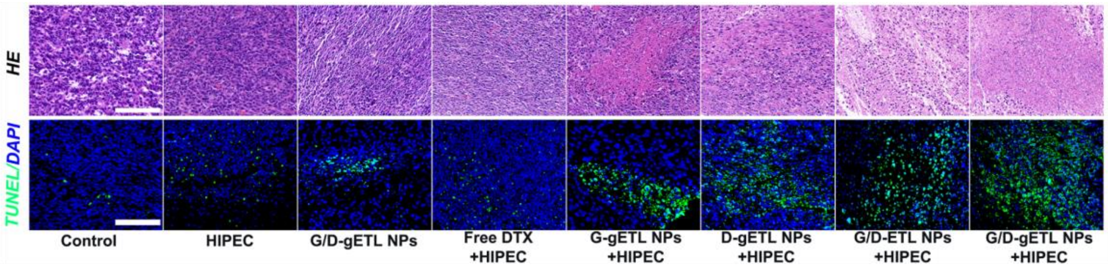

Figure S9. gETL NPs combined with HIPEC displayed synergistic anti-tumor effect (n=5). Mice were killed at the endpoint of observation (day30), the histological morphology of tumor was analyzed by hematoxylin-eosin staining of tissue sections, and the apoptosis of tumor cells was analyzed by TUNEL staining. Scale bar: 200 μm.

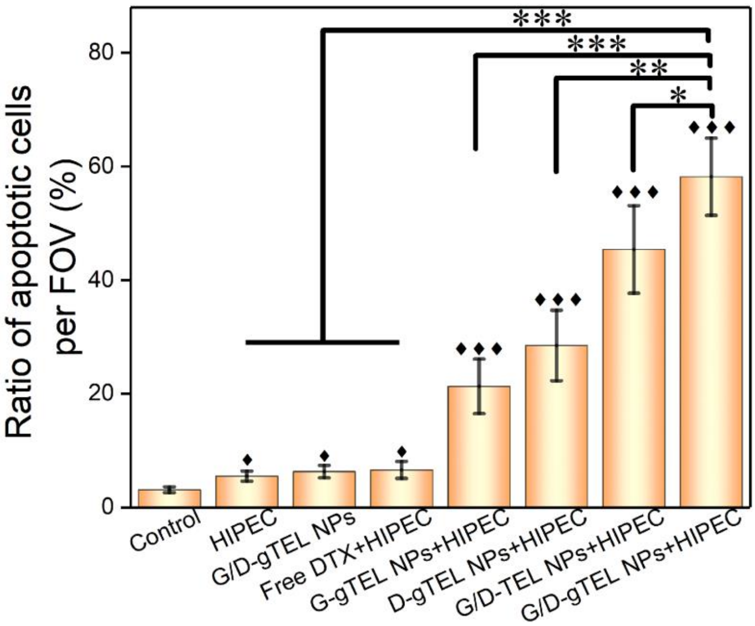

Figure S10. Semi-quantitative analysis of apoptosis of tumor cells in each group. Mean apoptotic rate from 5 high-power field per section was used for statistical difference analysis, One-way ANOVA was used to determine statistical differences. ♦, $p < 0.05$, ♦♦, $p < 0.01$, ♦♦♦, $p < 0.001$; compared with control group; *, $p < 0.05$; **, $p < 0.01$, ***, $p < 0.001$, compared with the indicated groups.

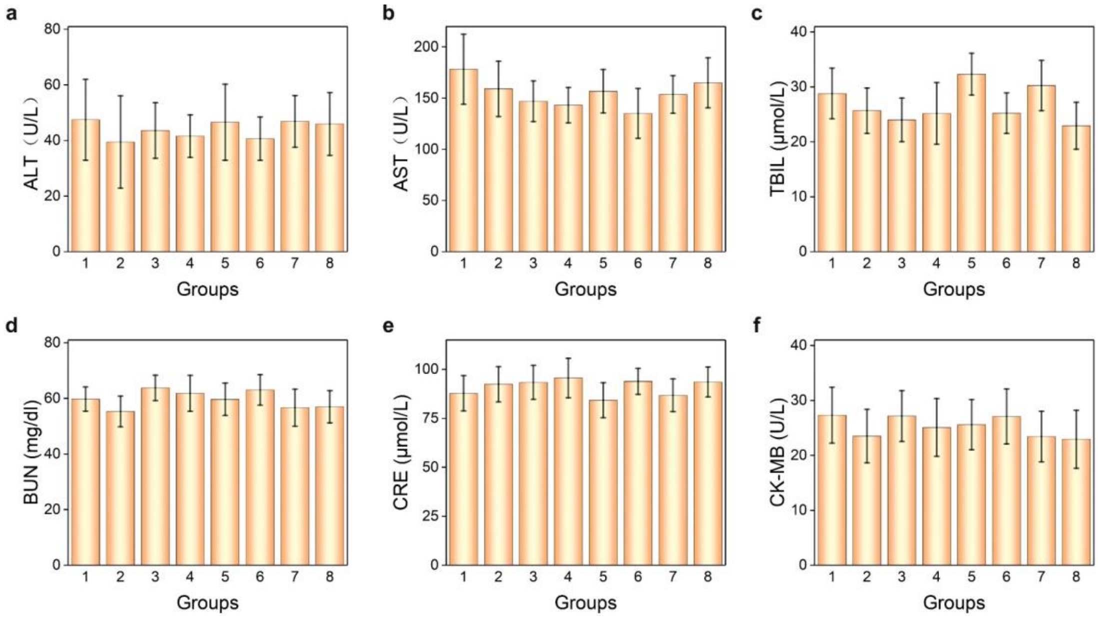

Figure S11. Both gETL NPs intravenous injection alone or combined with HIPEC displayed a satisfactory biological safety *in vivo* (n=5). Peripheral blood samples of animals were collected for biochemical analysis at the endpoint of observation (day 30). Biochemical indicators related to organ damage were detected using serum samples. **a)** ALT, glutamic-pyruvic transaminase. **b)** AST, glutamic-oxaloacetic aminotransferase. **c)** TBIL, total bilirubin. **d)** BUN, blood urea nitrogen. **e)** CRE, creatinine. **f)** CK-MB, Creatine Kinase-MB. **Group information**:**1** Control; **2** HIPEC only; **3** G/D-gETL NPs; **4** Free DTX+ HIPEC; **5** G-gETL NPs + HIPEC; **6** D-gETL NPs + HIPEC; **7** G/D-ETL NPs + HIPEC; **8** G/D-gETL NPs + HIPEC.

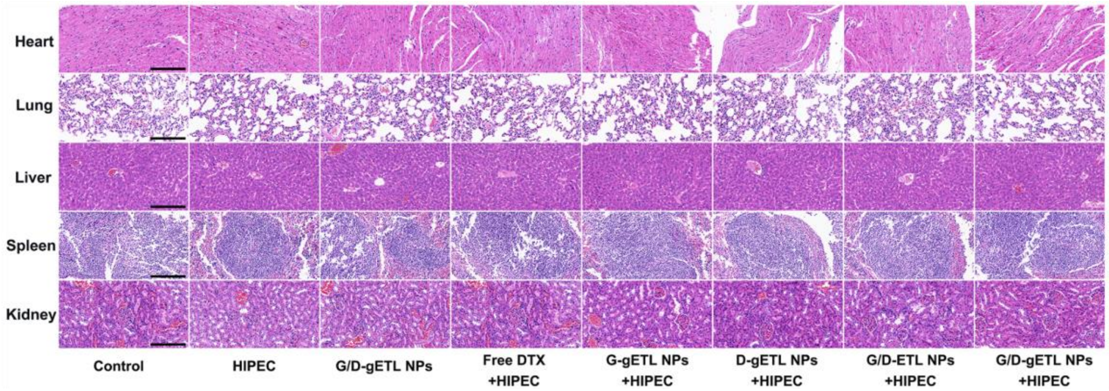

Figure S12. gETL NPs combined with HIPEC displayed anti-tumor effect with a satisfactory biological safety (n=5). Mice were killed at the endpoint of observation (day30), histological morphology of major organs (heart, lung, liver, spleen, and kidney) was analyzed by hematoxylin-eosin staining of tissue sections. Scale bar: 200 μm.

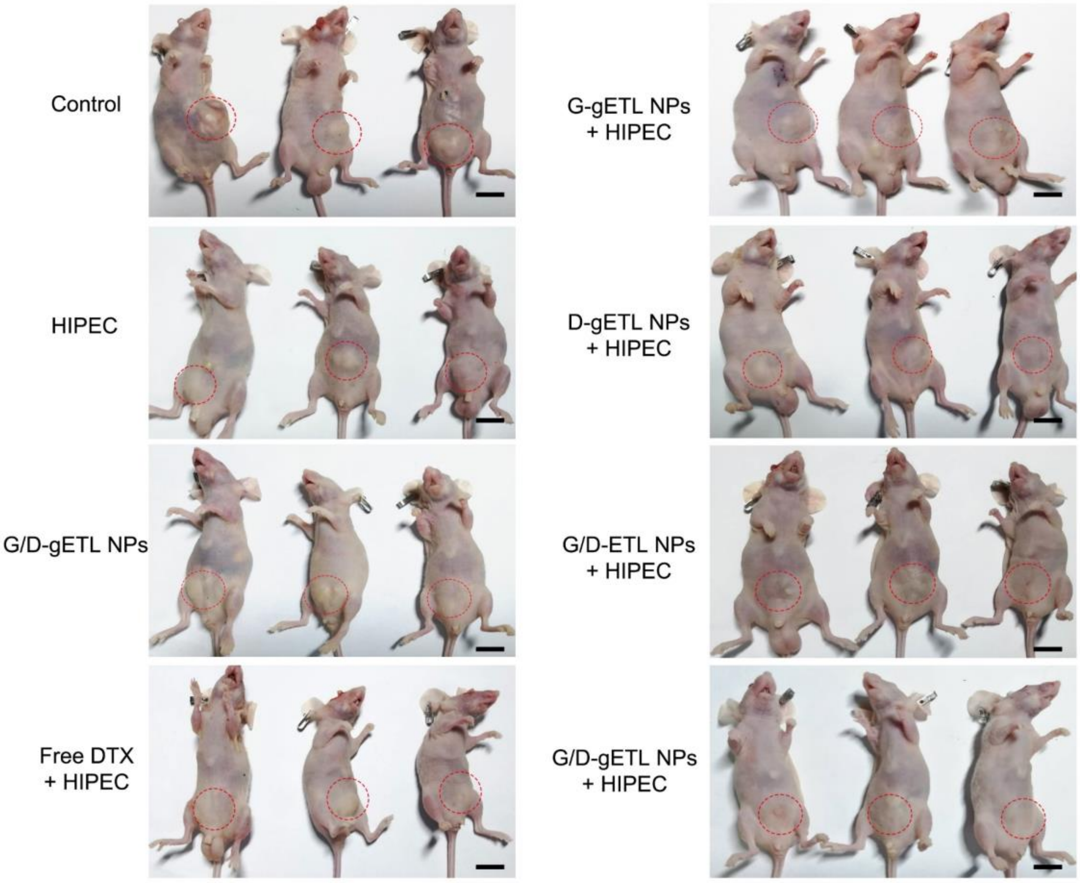

Figure S13. Therapeutic effects of NPs and HIPEC in PDX model of mPC (n=3). The tumor burden of each mouse at the endpoint of observation (day 30). Scale bar: 1 cm.

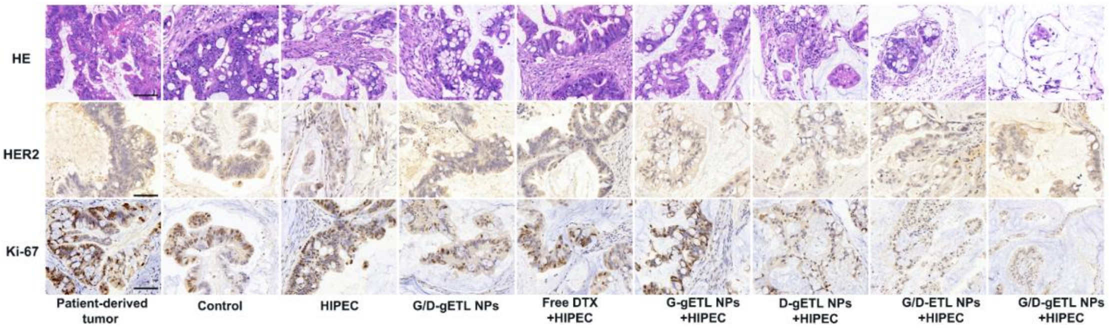

Figure S14. Therapeutic effects of NPs and HIPEC in PDX model of mPC. Mice were killed at the endpoint of observation (day 30), histological morphology of tumor sections was analyzed by hematoxylin-eosin staining **(HE)**. Immunohistochemical staining of **HER2** and **Ki-67** was used to identify homology between animal tumor and primary tumor derived from patients. Scale bar: 100 μm.

Materials and methods

Materials

The phospholipids including 1,2-dipalmitoyl-sn-glycero-3-phosphocholine (DPPC), 1-stearoyl-2-hydroxy-sn-glycero-3-phosphocholine (MSPC) and 1,2-distearoyl-sn-glycero-3-phosphoethanolamine-*N*-[methoxy(polyethylene glycol)-2000] (DSPE-PEG_{2000}) were purchased from A.V.T. Pharmaceutical Co., Ltd. (Shanghai, China). Docetaxel and oxaliplatin were purchased from Sigma Aldrich (Shanghai, China). Phosphate buffer saline (PBS), Dulbecco's modified Eagle medium (DMEM), Roswell Park Memorial Institute 1640 medium (RPMI 1640 medium) and fetal bovine serum (FBS) were purchased from Thermo Fisher Scientific Inc. (Shanghai, China). Exosome Starting Kit CD 9 and μColumns with plungers were purchased from Miltenyi Biotech Corporation (Germany). The Annexin V-fluorescein isothiocyanate (FITC) / propidium iodide (PI) apoptosis kit, 10% Bis-Tris gel and chemiluminescent kit were purchased from Beyotime Institute of Biotechnology (Guangzhou, China). PVDF membrane was purchased from Millipore (China) Co., Ltd. (Shanghai, China).

The primary antibodies anti-CD63, anti-CD9, anti-TSG101, anti-actin, and the secondary antibodies conjugated with horseradish peroxidase (HRP) were purchased from system biosciences Inc. (SBI) (California, USA). Radio immunoprecipitation assay (RIPA) lysis buffer was purchased from Solarbio Life Science (Guangzhou, China). Anti-HER2 and anti-Ki67 for human were purchased from Abcam (Shanghai) Trading Co., Ltd. (Shanghai, China). Anti-CD68-FITC, anti-CD86-PE, anti-CD206-FITC, anti-CD206-PerCP-Cy5.5, anti-CD3, anti-CD4 and anti-CD8 antibodies were purchased from BioLegend, Inc. (San Diego, USA). HRP-conjugated secondary antibodies, CY3, FITC, and CY5 were purchased from Servicebio technology CO.,LTD (Wuhan, China). ELISA kits for GM-CSF, TNFα, and IFN-γ analysis were purchased from Scissors gene Co., Ltd (Shenzhen, China). Other reagents and solvents such as 4',6-diamidino-2-phenylindole (DAPI),

1,1'-dioctadecyl-3,3,3',3'-tetramethyl indotricarbocyanine Iodide (Dir), chloroform, methanol, and Dimethyl sulfoxide (DMSO) were purchased from Sigma-Aldrich (Shanghai, China).

Human tissues, cell lines, and animals

The tumor tissues which were used to construct PDX animal models were obtained from mPC patients from general surgery department of the Third Affiliated Hospital of Sun Yat-sen University. Before the samples were taken, the patients' written consent was obtained and the experiments were approved by the Medical Ethics Committee of the Third Affiliated Hospital of Sun Yat-sen University.

Human colorectal cancer cells HCT116, human normal colon epithelial cells HCoEpic, mouse macrophages RAW264.7, mouse embryonic fibroblasts (BALB/c 3T3), and mouse colorectal cancer cells CT26 were purchased from the cell bank of Chinese Academy of Sciences (Shanghai, China). DMEM or RPMI 1640 medium containing 10% FBS and 1% penicillin/streptomycin were used for cell culture. All cells were cultured in humidified incubator at conditions of 37 °C, 5% CO_2 and 95% humidity.

Healthy, male, 5-week old BALB/c mice weighed at 17.52 ± 2.01 g and 5-week old BALB/c nude mice weighed at 18.12 ± 2.24 g were provided by Medical Experimental Animal Center of Guangdong Province. All animal experiments were performed under the guidelines approved by the Institutional Animal Care and Use Committee, Sun Yat-Sen University.

Construction of mPC animal models

To construct patient derived xenografts (PDX) animal models of mPC [1], tumor tissues that were obtained from mPC patients were cut into tissue fragments at size of 20 mm^3 and transplanted into the peritoneal cavity of BALB/c nude mice as soon as possible. For animal surgery, a 5 mm incision was performed on the middle abdomen of each anesthetized mouse. Then the tumor fragments were seeded into the lower

peritoneal cavity, and the incision was closed by 6–0 nylon surgical sutures (Ethicon Inc., USA). The passage and amplification of tumors between mice were implemented through surgery as previously described, and the third generation of PDX model mice were used for experiment. The expression of HER2 and Ki-67 in tumors that obtained from animals was detected by human antibodies and compared with primary patient-derived tumors to confirm the homology.

To construct cells-derived xenografts (CDX) animal model of mPC [2], each BALB/c mouse was intraperitoneally injected with 1×10^6 CT26 cells in logarithmic growth phase of.

HIPEC procedure

HIPEC procedure was performed in animals as reported [2]. Before operation, animals received inhalation anesthesia by 3% isoflurane with oxygen. Then, to build inflow and outflow channels, puncture needles were inserted into the left upper and the right lower abdomen, respectively. After connecting all pipes and setting the HIPEC equipment correctly, oxaliplatin was mixed with 100 mL 5% glucose solution and then added into the micro-perfusion system for HIPEC treatment for 1 hour, and the temperature of perfusion solution was controlled at 42 ℃ during the whole process. In order to prevent perioperative infection, the needle was removed after treatment and the puncture wounds were sutured. The operations were completed in the super clean platform at specific pathogen free (SPF) animal laboratory center.

Evaluation of drug delivery efficiency of HIPEC

To evaluate the drugs delivery efficiency of HIPEC, a CT26 cell-derived mPC model was constructed using BALB/c mice. Specifically, BALB/c mice were intraperitoneally injected with 1×10^6 CT26 cells. 10 days later, the ascites of mice were removed by abdominal puncture, then animals were randomly divided into 3 groups and treated with warm water intraperitoneally perfusion, 150 mg/m^2 Pt-based HIPEC, or 75 mg/m^2 Pt-based HIPEC, respectively. Animals were executed in 30 min

after post-treatment of HIPEC, and then tumor nodules with different volumes (< 10 mm^3 or >10 mm^3) were separately prepared into single cell suspensions. Platinum of each single cell was detected by single-cell ICP-MS (NexION 2000, PerkinElmer).

Preparation of thermosensitive liposomes

The thermosensitive liposomes were prepared by membrane hydration method as reported [3]. Briefly, lipids with the ratio of DPPC/DSPE-PEG_{2000}/MSPC at 90:10:4 (mol/mol/mol) were dissolved in chloroform for 30 min, and organic solvents were removed by rotary evaporator at 55 °C and vacuum conditions. The lipid film was washed by 10 ml PBS at 65 °C for 2 h, then the samples were subjected to ultrasound at 4°C for 10 min and subsequently filtered by polycarbonate membrane with pore size of 100 nm for 3 times. To prepare docetaxel-loaded thermosensitive liposomes, docetaxel was added into the lipid solutions with ratio of drug / lipids at 1:15 (w/w), and the remaining procedures were consistent with the steps of blank liposomes preparation. After filtered by polycarbonate membrane, the samples were added into 3500 kDa dialysis bags and dialyzed in PBS for 8 hours to remove the free drugs.

Gene transfection

Three kinds of transfection expression vectors to achieve over-expression of CD47, GM-CSF and CD47&GM-CSF were acquired from VectorBuilder Inc. (Guangzhou, China). The plasmid identities were validated by sequencing and the different plasmids were packaged by lentiviruses. Then mouse embryonic fibroblasts (BALB/c 3T3) were transfected with lentiviruses [4]. Specifically, 3T3 cells with a cell density of 1×10^4 cells per well were planted in 12-well culture plate for 12 h, then lentiviruses were added into fresh mediums with dose at multiplicity of infection (MOI) of 10, and 10 μg/ml polybrene was also added into the transfection system. 12 hours later, culture medium was refreshed and cells were cultured for 48 hours. Transfected cells were screened out by streptomycin and cultured in the medium that was prepared by exosome-free FBS.

Purification and characterization of engineered exosomes

The purification and characterization of engineered exosomes were performed according to reported [5]. About 1× 10^7 fibroblasts was cultured by 30 ml exosome-free medium for 72 h, and the medium was collected and centrifuged at 5,000 × g for 60 min at 4 °C to remove cell debris. The supernatant was filtered by 0.22-μm filters, and then concentrated by ultrafiltration using 100 kDa Centrifugal Filter Unit (UFC910096, Millipore). Then the resulting sample was centrifuged at 100,000 × g for 90 min at 4°C by a 45Ti rotor in an ultracentrifuge (OptimaTm L-100XP, Beckman Coulter). The precipitate at the bottom of tube was resuspended by PBS and centrifuged again at 120,000 × g for 70 minutes. After removing supernatant, exosomes were suspended by PBS for subsequent tests. The concentration of exosomes was measured by BCA kit (23225, Thermo Fisher). The morphology of exosomes was observed by transmission electron microscopy (TEM) (HT7700, Hitachi). After fixed by 2% paraformaldehyde, the samples were loaded onto a copper grid with carbon support film (FF200-Cu; Electron Microscopy Sciences) and stained negatively with sodium phosphotungstate solution according to the manufacturer's scheme. Electron microscopic images were obtained by TEM. Samples were diluted by PBS to the appropriate concentration, the particle size and concentration of exosomes were detected by Flow Nano Analyzer (N30) (Nano FCM Inc., Xiamen, China).

Western blot analysis

The samples were separated on 10% Bis-Tris gel by electrophoretic (Bio-Rad) electrophoresis and transferred to 0.45 μm PVDF membrane. The membranes were sealed in 10% (w/v) skimmed milk powder (NC9022655; Thermo Fisher) at 37 °C for 2 h and incubated with primary antibodies overnight at 4 °C. Then membranes were washed by 0.2% Tween-20 containing PBS for 3 times. After incubated with secondary antibodies for 2 hours and washed 3 times, the membranes were developed with chemiluminescent reagents.

ELISA

The concentrations of GM-CSF in the fibroblasts supernatants, fibroblasts derived exosomes and NPs were determined by murine GM-CSF ELISA kit [4]. The concentrations of TNF-α and IFN-γ in tumor tissue homogenate and serum compared with control group were determined by murine TNF-α and IFN-γ ELISA kit.

For cell supernatants, tissue homogenate and serum samples, samples were centrifuged for 20 minutes at 1,000×g and the supernatants were collected for ELISA test according to the manufacturer's protocol. For exosomes and NPs, the protocol was slightly modified. Briefly, samples were ultra-centrifuged at 120,000 × g for 70 minutes, and the sediments were collected and quantified by BCA or weighed after freeze-drying. For test, exosomes or NPs were pre-treated in 100 μL of PBS with 0.05% Tween-20 for 30 min to break the membranes at room temperature, then samples were added into the plate that pre-coated with capture antibodies and incubated at 37 °C for 1h, then the plate was washed by PBS. After incubated with detection antibodies for 1 h and avidin-HRP, the absorbance at 450 nm was measured by Power Wave XS (BioTek), the concentration of each sample was calculated according to the standard curve.

Preparation and Characterization of NPs

NPs were prepared based on thermosensitive liposomes and engineered exosomes by membrane fusion technology using freeze–thaw method as reported.[6, 7] Briefly, docetaxel-loaded liposomes and engineered exosomes were rapidly mixed at ratio of 1:1 (mol / mol) and incubated at 37 °C for 30 min, then the samples were frozen by liquid nitrogen for 5 min and thawed at room temperature for 15 min. After 3 freeze–thaw cycles were repeated, the hybrid NPs were formed via the lipid membrane fusion between the liposomes and the exosomes. In order to remove the free drugs, the samples were added into 3500 kDa dialysis bags and dialyzed in PBS for 8 h. To determine the presence of CD47 on the surface of NPs, sample was incubated with FITC-CD47 antibody at 37 °C for 30 min, 10-fold volume of PBS was added into the

sample, then 100 kDa ultrafiltration tube was used to filter and concentrate the sample to original volume, 3 cycles of ultrafiltration were repeated to remove the free antibodies, then the sample was detected by Flow Nano Analyzer.

Evaluation of membrane fusion between exosomes and liposomes

The membrane fusion between liposomes and exosomes was confirmed by a two-way verification. Firstly, NBD-labeled liposomes were prepared by NBD-DSPE-PEG2000, and exosomes were labeled by CD9 immunomagnetic beads, then liposomes and exosomes were mixed with each other at ratio of 1:1(mol/mol). Then the mixture was extracted in magnetic field and unfused liposomes were removed. Finally, samples including exosomes, liposomes, and mixture were observed by TEM. Samples were also used to treat HCT116 cells for 2 h and the fluorescence was observed by CLSM.

In order to quantitatively evaluate the fusion efficiency, the methods of immunomagnetic separation and FITC-labeled lipids for nanoflow analysis was adopted. In short, similar to previous two-way validation experiment, after fusion process, the mixtures containing hybrid vesicles and unfused exosomes were sorted by the immunomagnetic beads, then the fluorescence signal and particle counts of fused hybrid NPs was detected by Flow NanoAnalyzer. According to the accurate count results of nanoparticles, the efficiency of membrane fusion between exosomes and liposomes was evaluated.

Thermosensitivity of gETL NPs *in vitro*

The drug release behavior of NPs at different temperature in PBS with 10% FBS was determined by dialysis method. Briefly, 6 mg NPs were suspended in 5 mL solution (PBS with 10% FBS) and added into 3000 Da dialysis bags, then dialysis bags were put in 30 mL solution and stirred with 100 rpm at 37 °C, 39 °C, 41 °C, 42 °C, respectively. At the time points of 1, 2, 3, 4, 5, 10, 20, 30, and 60 min, 0.5 mL

of sample solution was obtained and equal amount of fresh solution was added into the system. Then the docetaxel content in each sample was detected by HPLC.

Cytotoxicity of gETL NPs

To test the cytotoxicity of blank vectors, CT26 cells or HCoEpic cells were seeded in 96-well plates and incubated with PBS, wild type exosomes, engineered exosomes, blank liposomes, and blank gETL NPs at various concentrations at 37 °C for 12 h. Then the cell viability was determined by MTT assay. Next, the anti-tumor effects of free gETL NPs or combined with oxaliplatin thermo-chemotherapy were evaluated comprehensively. Briefly, 1×10^4 cells per well were plated in 96-well plates and cultured for 12 h, then samples were treated by NPs with different cargos for 2 h, 5 mg/ml oxaliplatin was added into each well, and samples were incubated at 42 °C for 1 h to simulate the working conditions of HIPEC. Then plates were transferred into 37 °C incubator and incubated for another 8 hours, MTT assay was used to detect the cell viability of each sample. Flow cytometry was used to determine the apoptosis of cancer cells in each group. Briefly, after the treatments as described above, the cancer cells were stained by Annexin V-FITC/PI apoptosis detection kit according to the manufacturer's suggestion. Then the samples were detected by flow cytometry (FC500, Beckman Coulter, Fullerton, CA, USA).

gETL NPs regulated repolarization of macrophages from M2 to M1 phenotype *in vitro*

To evaluate the regulation of macrophages repolarization of gETL NPs, 1×10^5 RAW264.7 macrophages were plated and stimulated by IL-4 (40 ng/ml) for 24 hours to induce M2 polarization, then the supernatant was removed and replaced with fresh medium, gETL NPs (80 μg/ml, equivalent to free 0.5 ng/ml GM-CSF) with different cargos or free GM-CSF (0.5 ng/ml) were added into the medium and incubated for another 24 hours, and PBS was used as the control group. Then cells were stained with anti-CD206-FITC and anti-CD86-PE and detected by CLSM (FV3000, Olympus,

Japan). For quantitative analysis, cells were stained with anti-CD68-FITC, anti-CD86-PE, anti-CD206-PerCP-Cy5.5 and detected by flow cytometry.

gETL NPs promoted M1 macrophages phagocytosis of cancer cells

To determine the effect of CD47 modified gETL NPs on the M1 macrophages phagocytosis of cancer cells, RAW264.7 macrophages were plated and incubated with 0.5 ng/ml GM-CSF for 24 hours to be induced to M1 macrophages. And then ETL NPs, gETL NPs and CD47 protein were added into wells and incubated for 2 hours, PBS was used as the control. Moreover, mCherry overexpressed HCT116 cells were constructed by gene transfection and used as model cells. The adherence ability of HCT116 cells were damaged by 50 μg/ml oxaliplatin. Then these HCT116 cells were collected and added into each well at the quantity ratio of 1:5 (macrophages/cancer cells) and incubated for 6 hours. Then plates were washed by PBS three times to remove free cancer cells, and samples were detected by confocal laser scanning microscopy (CLSM). For quantitative analysis, samples were prepared into single cell suspension and detected by flow cytometry.

To explore the possible mechanism of gETL NPs promoting M1 macrophages phagocytosis of cancer cells via CD47-SIRPα signal axis. RAW264.7 macrophages were incubated with 0.5 ng/ml GM-CSF for 24 hours to be induced to M1 macrophages, and then ETL NPs, gETL NPs and CD47 protein were added into wells and incubated for 2 h, PBS was used as control. Then anti-SIRPα-FITC antibodies were added into the medium directly and incubated for 30 min, followed by being washed by PBS for three times, the samples were stained by DAPI and detected by CLSM. The images of SIRPα molecule exposure on macrophages of each group were captured and calculated by ImageJ software.

***In vivo* biodistribution of gETL NPs**

In order to study the biodistribution of gETL NPs, PDX model of mPC in BALB/c nude mice was constructed by using patient-derived xenograft, and Dir was loaded

into liposomes, ETL NPs and gETL NPs, respectively. After the tumor volume reached to 50 mm^3, animals were intravenously injected with liposomes, ETL NPs, gETL NPs at an identical Dir dose of 2.5 mg/kg. Then animals were monitored by In Vivo Imaging System (IVIS) at 2 h, 4 h, 8 h, 12 h, 24 h, and 48 h post-injection. After euthanasia at 48 h post-injection, the animals were dissected and organs were exposed under IVIS. Then animals were sacrificed, the tumor and major organs (heart, lung, liver, spleen, intestine, and kidney) were harvested for *ex vivo* imaging immediately. The biodistribution of liposomes, ETL NPs, or gETL NPs in the major organs and tumor at 48 h post-injection was analyzed in terms of fluorescence intensity. The Proportion of fluorescence distributed in each organ and tumor at 48 h post-injection was also analyzed.

Pharmacokinetics of gETL NPs

To evaluate pharmacokinetics of gETL NPs, BALB/c mice were intravenously injected with free docetaxel, docetaxel-liposomes, docetaxel-ETL NPs, docetaxel-gETL NPs at an identical docetaxel dose of 5 mg/kg. Then, 3 mice in each group were anesthetized to obtain blood samples at different time of 0.5, 1, 2, 4, 8, 12, 24, and 48 hours of post- injection, respectively. The blood samples were centrifuged at 2000 g for 15 min at 4 °C to prepare the plasma, and the concentration of docetaxel in plasma was detected by HPLC.

Anti-tumor activity of gETL NPs combined with HIPEC in animal models

The anti-tumor effect of combination therapeutic that based on HIPEC and NPs was evaluated in PDX and CT26-derived CDX animal models of mPC. For CDX model, animals were randomly divided into 8 groups at 10 days post-injection (n = 12). The detailed grouping information includes: 1) Control ; 2) HIPEC only; 3) G/D-gETL NPs *i.v.* only; 4) free docetaxel *i.v.* + HIPEC; 5) G-gETL NPs *i.v.* + HIPEC; 6) D-gETL NPs *i.v.* + HIPEC; 7) G/D-ETL NPs *i.v.* + HIPEC; 8) G/D-gETL NPs *i.v.* + HIPEC.

After grouped, the ascites of each mouse was removed by abdominal puncture, and the volume of ascites was measured as ascites baseline. Then free docetaxel or NPs was injected through tail vein at an identical docetaxel dose of 1.5 mg/kg immediately. And 12 hours later, 75 mg/m^2 oxaliplatin was dissolved in 100 mL 5% glucose solution and added into the micro-perfusion system and performed HIPEC for 1 hour, and the temperature of perfusion solution was controlled at 42 °C during the whole process. The 2nd same treatment was performed at day 17. The body weight and abdominal circumference of mice were measured every 3 days. With two weeks of continuous observation, 5 animals of each group were euthanized at day 30 post-injection for anti-tumor analysis, the rest of the animals (n = 7) were continued to be observed for survival analysis. For anti-tumor analysis, mice were sacrificed, and the blood samples, ascites, tumors and major organs were obtained. The ascites volume was measured and compared with ascites baseline, and the tumor burden was evaluated by using experimental peritoneal carcinomatosis index (PCI) system.

For PDX model, patient-derived mPC (originated from colorectal cancer) tissues were cut into fragments and implanted on the peritoneum of the lower abdomen of mice. At day 10 of post-implantation, animals with tumor volume at about 50 mm^3 were randomly divided into 8 groups (n = 3), the detailed grouping information as followed: 1) Control ; 2) HIPEC only; 3) G/D-gETL NPs *i.v.* only; 4) free docetaxel *i.v.* + HIPEC; 5) G-gETL NPs *i.v.* + HIPEC; 6) D-gETL NPs *i.v.* + HIPEC; 7) G/D-ETL NPs *i.v.* + HIPEC; 8) G/D-gETL NPs *i.v.* + HIPEC. At day 10 and day 17, NPs with different cargos were injected via tail vein and the oxaliplatin-based HIPEC treatment was given 12 h later, then animals were under observation to day 30 of post-implantation. And mice were sacrificed at day 30, the blood samples, tumors, and major organs were obtained for analysis.

Evaluation of tumor burden by Peritoneal carcinosis index (PCI)

To evaluate the tumor burden of animals, PCI evaluation system that based on sizes and distributions of tumor, and ascites was adapted from literature report [2]. Briefly,

the abdominal cavity was divided into four regions anatomically: region I for sub-diaphragm; region II for the surface of liver, spleen, stomach; region III for the surface of small intestine, colon, urogenital system, rectum and mesenterium; region IV for the surface of abdominal wall. The tumor burden of each region was scored according to the greatest diameter of the nodules. The scoring criteria was set as follows: score 0 for no tumor nodules; score 1 for nodule size ≥ 2 mm; score 2 for nodule size ≥ 2 mm and up to 5 mm; score 3 for nodule size ≥ 5 mm and up to 10 mm; score 5 for nodule size ≥ 10 mm. And another score 1 for appearance of ascites, the sum of all score was the PCI score for each animal (ranging from 0 to 21).

Analysis of hematological and histological

To explore hematological and histological analysis, blood samples were collected at day 30 and centrifuged at 2000 g for 15 min, then serum samples were tested for biochemical markers. All tumors and major organs were fixed by 4% paraformaldehyde, then tumors were dissected and sectioned for TUNEL staining, H&E staining, immunohistochemical staining, and immunofluorescence staining. Organs including heart, lung, liver, spleen, and kidney were dissected and sectioned for H&E staining. Hematological and histological analysis were carried out by Servicebio (Wuhan, China).

Analysis of immune response *in vivo*

Blood samples were collected at day 30 and centrifuged at 2000 g for 15 min to collect serum samples. Moreover, tumors were weighed and prepared into tissue homogenate. The concentrations of TNF-α and IFN-γ in serum and homogenate were analyzed by ELISA. Markers including CD68, CD86, and CD206 were detected by immunofluorescence staining of tumor sections to evaluate the polarization of macrophages in tumor microenvironment. T cells infiltration in tumor tissue was evaluated by immunofluorescence staining of CD3, CD4, and CD8 of tumor sections.

The images were calculated by ImageJ software, and positive cells from 5 high-power field per section were used for statistical analysis.

Statistical analysis

The SPSS 22.0 software was used for statistical analysis. Data were presented as mean ± standard deviation (mean ± S.D). Student′s *test* was applied for comparing the difference between two groups while a one-way analysis of variance (ANOVA) was applied for comparing the difference among multiple groups. $p < 0.05$ was considered be statistically significant between groups.

Reference

1. Y. Hiroshima, Y. Zhang, N. Zhang, A. Maawy, S. Mii, M. Yamamoto, F. Uehara, S. Miwa, S. Yano, T. Murakami, M. Momiyama, T. Chishima, K. Tanaka, Y. Ichikawa, M. Bouvet, T. Murata, I. Endo, R. M. Hoffman, *Plos One* **2015**, *10*, e0117417.
2. L. H. Shao, S. P. Liu, J. X. Hou, Y. H. Zhang, C. W. Peng, Y. J. Zhong, X. Liu, X. L. Liu, Y. P. Hong, R. A. Firestone, Y. Li, *Cancer-Am Cancer Soc* **2012**, *118*, 2986.
3. X. L. Liang, J. Gao, L. D. Jiang, J. W. Luo, L. J. Jing, X. D. Li, Y. S. Jin, Z. F. Dai, *Acs Nano* **2015**, *9*, 1280.
4. K. Yaddanapudi, S. H. Meng, A. G. Whitt, N. Al Rayyan, J. Richie, A. Tu, J. W. Eaton, C. Li, *Oncoimmunology* **2019**, *8*, 1561119.
5. S. Kamerkar, V. S. Lebleu, H. Sugimoto, S. J. Yang, C. F. Ruivo, S. A. Melo, J. J. Lee, R. Kalluri, *Nature* **2017**, *546*, 498.
6. Y. T. Yang, G. X. Shen, H. Wang, H. X. Li, T. Zhang, N. J. Tao, X. T. Ding, H. Yu, *Proc. Natl. Acad. Sci. USA* **2018**, *115*, 10275.
7. Y. T. Sato, K. Umezaki, S. Sawada, S. Mukai, Y. Sasaki, N. Harada, H. Shiku, K. Akiyoshi, *Sci Rep* **2016**, *6*, 21933.
